# Immunological and Clinical Outcomes Associated with Histotripsy Ablation in Spontaneously Occurring Canine Osteosarcoma; Veterinary Clinical Trial Results

**DOI:** 10.3390/cancers18152511

**Published:** 2026-08-05

**Authors:** Alayna. N. Hay, Lauren Ruger, Elliana R. Vickers, Sheryl Coutermarsh-Ott, Eli Vlaisavljevich, Joanne Tuohy

**Affiliations:** 1 Department of Small Animal Clinical Sciences, Virgina Maryland College of Veterinary Medicine, Blacksburg, VA 24061, USA; ellianarv@vt.edu (E.R.V.); jltuohy@vt.edu (J.T.); 2 Virginia Tech Animal Cancer Care and Research Center, Virginia Maryland College of Veterinary Medicine, Roanoke, VA 24016, USA; 3 Department of Biomedical Engineering, Virginia Tech, Blacksburg, VA 24061, USA; larnold24@vt.edu (L.R.); eliv@vt.edu (E.V.); 4 Translational, Biology, Medicine and Health Graduate Program, Roanoke, VA 24016, USA; 5 Department of Biomedical Sciences and Pathobiology, Virginia Maryland College of Veterinary Medicine, Blacksburg, VA 24061, USA; slc2003@vt.edu

**Keywords:** bone cancer, comparative oncology, mechanical focused ultrasound, dog, novel therapy

## Abstract

Background: Osteosarcoma (OS) is a devasting bone cancer that occurs primarily in pediatric patients, and pet dogs, and treatment options have not advanced in decades. There is a profound need for advancement of treatment options to improve patient prognosis given the grim 70% 5-year survival rate for human patients, and 10–12-month median survival for canine patients. A major hurdle in advancing treatment options is overcoming the immunosuppressive tumor microenvironment to mitigate metastatic disease progression—the leading cause of mortality in OS patients. Methods: We investigated the ability of a mechanical focused ultrasound ablation modality, histotripsy, to induce acute immunomodulation in canine patients with spontaneously occurring OS. Results: Immunomodulation was evident locally and systemically. Tumor gene signatures indicated upregulation of pro-inflammatory signaling pathways and T-cell receptor signaling, and B-cell activation, and systemically, increased monocyte activation was observed within 5 days post-histotripsy ablation. A subset of patients who received histotripsy prior to definitive standard-of-care exceeded the reported expected median survival time of 10–12 months for canine OS patients that received definitive standard-of-care only. Conclusions: Our results demonstrate the promising potential of histotripsy to overcome the immunosuppressive tumor microenvironment.

## Simple Summary

Osteosarcoma (OS) is a devasting bone cancer that affects children and adolescents, and pet dogs. Unfortunately, OS is an aggressive disease and the survival outcomes for both human and pet dog patients are not favorable. To improve patient outcomes, novel treatment options are profoundly needed. To develop novel treatment options, researchers can investigate OS in pet dogs and translate the findings to humans due to the similarities in the biology of OS in humans and dogs. In this study we investigated a novel treatment modality called histotripsy which uses mechanical high intensity focused ultrasound to target and destroy cancer cells. We found that histotripsy may upregulate the body’s cancer defense system (i.e., the immune system), which is essential for an effective cancer treatment option. The findings from our study suggest that further development and investigation of histotripsy ablation for treating OS would be beneficial.

## 1. Introduction

Cancer is a devasting disease resulting in millions of deaths worldwide annually, in both human and canine populations [1]. Osteosarcoma (OS) is one of several types of cancers that occur in both humans and dogs. It is the most frequently occurring primary bone malignancy in both pediatric and canine cancer patients, accounting for greater than 55% and 80–90% of primary bone tumor diagnoses, respectively [2,3,4]. Human and canine OS share similar biological behaviors [5,6,7], global gene expression signatures [7,8], and nearly identical histological characteristics [5,6,9]. These similarities allow pet dogs with spontaneously occurring OS to serve as a strong comparative oncology research model for human OS to bridge the translational gap from murine preclinical studies to human clinical trials. Current definitive standard-of-care treatment for human and canine OS includes surgical resection of the primary tumor via limb amputation or limb-salvage surgery and chemotherapy (neoadjuvant and adjuvant for humans, and only adjuvant for dogs) for management of metastatic disease. Both mentioned surgical techniques are invasive procedures with potential for complications and functional impairment [10,11,12,13,14,15]. In addition to the challenges of surgical primary tumor resection, metastatic disease remains the predominant cause of mortality despite chemotherapeutics [5,9,16]. The survival outcomes for OS patients have not improved significantly for decades. In the absence of metastatic disease at the time of OS diagnosis, the 5-year survival for human OS patients is ~70% and the expected median survival for dogs is 10–12 months [9].

In efforts to advance OS treatment options and improve prognosis, our team has investigated the use of a mechanical focused ultrasound ablation modality, histotripsy, for ablation of spontaneously occurring primary canine OS [17,18,19,20]. Histotripsy is a non-thermal, non-ionizing, and non-invasive focused ultrasound therapy currently under development for a variety of applications in human [21,22] and veterinary medicine [22,23]. We previously reported on the safety and feasibility of successfully employing histotripsy as a means to overcome the challenges associated with surgical resection of the primary tumor in canine bone cancer patients [17,18,20,24,25]. Our previous work demonstrated that partial tumor volume ablation with histotripsy is well tolerated, and ablation was evident histologically based on gross and microscopic observation at 1 day post-histotripsy (DPH) [20] and 3 and 5 DPH [25]. Additionally, prior work in preclinical rodent models, including an OS murine model, has shown that the destruction of cancer cells with histotripsy not only leads to the direct lysis of targeted tumor cells but can also alter the immunosuppressive TME through the release of tumor antigens and damage-associated molecular patterns (DAMPs) [19,26,27,28,29,30].

Often, an immunosuppressive TME allows for an environment that promotes the development of metastatic disease, and such is the case for OS [31,32,33]. Thus, modalities such as histotripsy which can induce immunomodulation and reprogram the TME to stimulate an anti-tumor immune response have high potential to mitigate metastatic disease and improve the survival prognosis for OS patients. Given the valuable role the immune response plays in improving OS patient prognosis and the immunomodulatory potential of histotripsy, the goal of the current study was to investigate the immunological outcomes associated with histotripsy ablation of canine primary OS. The results presented in this manuscript build on our prior work, which reports on the ablative outcomes associated with histotripsy ablation of canine OS [20,24,25]. The local and systemic immune responses and long-term clinical outcomes associated with histotripsy ablation were evaluated in a total of 28 dogs with primary OS and are presented in this current manuscript.

## 2. Materials and Methods

### 2.1. Patient Enrollment and Delivery of Histotripsy

The patients in this manuscript were enrolled into one of two veterinary clinical trials which were designed to evaluate the feasibility and safety of delivering histotripsy to partial tumor volumes to canine patients with spontaneously occurring bone tumors, and explore the immunological outcomes. The clinical trial criteria required limb amputation surgery 1 day post-delivery of histotripsy (clinical trial 1, n = 10 patients) or 3 or 5 days post-delivery of histotripsy (clinical trial 2, n = 11 patients for each of the two timepoints), and at the owner’s discretion the dog received adjuvant chemotherapy. For both clinical trials the canine patients were suspected to have OS based on cytological and/or radiographic evidence with no radiological evidence of pulmonary metastatic disease or prior tumor-directed therapy. The pooling of the two clinical trials was planned prospectively, and patients were treated with the same histotripsy parameters. The surgical timepoints were selected based on the pilot and safety nature of the trials. The allocation of dogs to the 1, 3 and 5 DPH surgical timepoints was determined relatively randomly with clinician and owner schedules, and the need to allocate at least 10 dogs to each group, being the only two contributing factors to timepoint selection. Prior to the delivery of histotripsy, CT scans were obtained to aid in the planning for histotripsy ablation and histotripsy was delivered to 2 cm^3^ volume of the tumor for all patients. Histotripsy was delivered under general anesthesia following standard-of-care protocols for client-owned dogs, and anesthesia was maintained using inhaled isoflurane during histotripsy treatments. Post-limb amputation surgery, all canine OS patients were followed long-term for evaluation of metastatic disease development and survival. Survival time post-histotripsy was calculated based on the time elapsed from the histotripsy treatment date to the date of reported death/euthanasia. The disease-free interval (DFI) was calculated based on the time between limb amputation surgery and development of metastatic disease. Additionally, to serve as controls for tumor immune evaluations, tumor samples were collected from OS patients who did not receive histotripsy ablation, or any tumor-directed therapy (treatment-naïve tumors) (Table S2). All experimental protocols were approved by the Virginia Tech Institutional Animal Care and Use Committee (IACUC) protocol (19-229). All methods were carried out in accordance with relevant guidelines and regulations. Informed consent was obtained from the owners.

### 2.2. Adverse Event Reporting

The Veterinary Cooperative Oncology Group Common Terminology Criteria for Adverse Events (VCOG-CTCAEv2) was used to report any adverse events relating to histotripsy ablation [34]. The most relevant categories for evaluation were cardiac arrhythmia, constitutional clinical signs, dermatology/skin, and musculoskeletal/soft tissue.

### 2.3. Surgical Resection of Tumor and Tumor Sample Collection

At 1, 3, or 5 days post-histotripsy ablation (DPH), the primary bone tumor was surgically resected via standard-of-care limb amputation surgery by a board-certified veterinary surgical oncologist (J.T.) at the Virginia Maryland College of Veterinary Medicine teaching hospital (Figure S1). Patients were discharged to their owners when considered appropriate by the attending clinician. Post-limb amputation surgery tumor samples were collected from the histotripsy ablated and non-ablated tumor regions by a board-certified veterinary pathologist (S.C.O). The histotripsy ablation region was identified by marking the skin overlying the ablated tumor region with ink. Additionally, the pathologist (S.C.O) was also informed of the region of the histotripsy targeted tumor (via verbal and/or written communication), and the project principal investigator (J.T.) was present at the time of gross sample collection when possible. Collected tumor samples were then stored in either RNAlater solution (ThermoFisher Scientific, Waltham, MA, USA), fixed in 10% formalin, or placed in DMEM culture media (ATCC, Manassas, VA, USA). Tumor samples were also collected from canine OS patients who had received no tumor-directed therapy (treatment-naïve) prior to limb amputation surgery to serve as a negative treatment control for immune evaluation. The samples from treatment-naïve tumors were collected under the same conditions as the patients that received histotripsy.

### 2.4. Blood Sample Collection

For all blood samples, immune evaluations results are only reported for OS patients. Supplemental Figure S1 depicts a schematic of tumor sample processing.

At the time of study enrollment, on the day of limb amputation surgery, and 2 weeks post-limb amputation surgery, 20 mL of blood was collected into EDTA vacutainers via venipuncture (Figure S1). Blood was collected from age- and weight-matched healthy dogs to serve as controls for OS patients. A healthy dog was defined as one not presenting with any clinical signs of ailment or disease and not receiving any immunomodulating medications. Owner consent was obtained prior to the blood draw. Blood was stored at 4 °C and processed within 24 h of collection.

### 2.5. Tumor Sample Processing

Given the focus of this manuscript, for all tumor immune evaluations, results are only reported for OS patients. Supplemental Figure S1 depicts a schematic of tumor sample processing.

#### 2.5.1. Histological Assessment

Formalin-fixed tissue samples collected post-limb amputation were paraffin-embedded, sectioned onto slides, and routinely stained with hematoxylin and eosin (H&E); tumor samples were evaluated for diagnostic purposes to confirm OS diagnosis and to assess cell viability and inflammation within the ablated tumor regions as previously described [20,25]. To build on our previous work [20,25], we compared cell viability and inflammation within histotripsy ablated tumor regions amongst the three groups (1, 3, and 5 DPH groups). For these group comparisons, cell viability within histotripsy ablated tumor regions was calculated based on the percentage of the tumor section exhibiting morphologic characteristics of dead or dying cells (cytoplasmic and nuclear fragmentation, cell shrinkage, nuclear pyknosis, cytoplasmic hypereosinophilia, and/or loss of distinct cell borders). The degree and distribution of inflammation was assessed separately for the bulk of the treated region. The degree of inflammation was scored qualitatively with a score of 0—indicating no significant inflammation, 1—indicating mild inflammation, 2—indicating moderate inflammation, and 3—indicating marked inflammation. For each tumor, one section containing one ablated region of tumor was evaluated. The evaluating pathologist was aware of whether histotripsy was delivered or if the sample was collected from a treatment-naïve dog.

#### 2.5.2. Differential Gene Expression

Paired histotripsy ablated and non-ablated regions of patient tumor samples that were stored in RNAlater at the time of collection were thawed, and total RNA was extracted from approximately 50 mg of tumor tissue. Tumor tissue was homogenized (Omni International, Kennesaw, GA, USA) in TRI Reagent (Zymo, Irvine, CA, USA), the homogenate was centrifuged at 12,000 *g* × 10 min and the Directzol total RNA extraction (Zymo, Irvine, CA, USA) protocol was followed according to the manufacturer’s recommendations. RNA with a 260/280 ratio of 1.8–2 was used for NanoString analysis with the Canine IO panel (Seattle, WA, USA). Absolute gene transcript count data was acquired on the nCounter Sprint instrument and analyzed using NanoString nCounter Data Analysis Software, nSolver (v4). The nSolver normalization method was based on assay-specific internal controls for background correction, positive control for hybridization efficiency, and housekeeping gene expression; differential expression criteria were based on comparing ablated tumor regions to non-ablated regions or ablated and non-ablated regions to treatment-naïve samples. Fold change threshold was set to < or >2, significance cutoff was set as *p* < 0.05 and *p*-values were adjusted based on FDR. For each NanoString reaction 100 ng of RNA was utilized and the probe hybridization step was conducted for 16 h. For gene enrichment analysis, KEGG and Gene Ontology biological pathways for characterizing gene sets were used [35]. A subset of 6 paired non-ablated and ablated tumor patient samples from each of the 3 histotripsy groups (1, 3 and 5 DPH) were randomly selected for the tumor differential gene expression analysis. Additionally, total RNA was extracted from treatment-naïve tumor samples (n = 5) to serve as controls for results interpretation.

#### 2.5.3. Tumor Immunophenotyping

Paired histotripsy ablated and non-ablated regions of tumors that were stored in DMEM media after collection were processed within 24 h post-collection for characterization of lymphocyte populations. A 0.5 cm × 0.5 cm portion of tumor samples was minced into 2–3 mm pieces, and then placed in a 15 mL conical tube with DMEM media (ATCC, Manassas, VA, USA) and an enzyme mixture of collagenase IV (20 mg/mL) and DNase I (10,000 Kunitz/mL), incubated at 37 °C for 60 min, strained through 40 µm and 70 µm filters again to remove undigested tumor fragments, centrifuged at 200 *g* × 10 min, and resuspended in buffer appropriate for flow cytometry. A total of 1 × 10^6^ cells was used for each flow cytometry sample. The following primary antibodies were used for lymphocyte phenotyping: CD5 (clone YKIX322.3), CD4 (clone YKIX302.9), CD8 (clone YCATE55.9), and CD21 (clone LT21) and eFluor 506 fixable viability dye was used for viability assessment. The CD4 was purchased from BioRad (Hercules, CA, USA); CD21, CD8, CD5, and viability dye were purchased from Invitrogen (Waltham, MA, USA). Prior to staining with primary antibodies, the PBMCs were stained with canine-specific Fc inhibitor (polyclonal, Invitrogen, Waltham, MA, USA) following the manufacturer’s protocol. All primary antibody staining was conducted as follows: 1 × 10^6^ cells/100 µL were stained with the primary antibodies by incubating for 30 min at 4 °C in the dark, followed by a single wash step (cells pelleted via centrifugation at 300 *g* for 6 min) to remove unbound excess antibodies, and analyzed on CytoFLEX cytometer (Beckman Coulter, Brea, CA, USA). Data analysis was conducted with Kaluza software (V2.2, Beckman Coulter) based on the single live cell population; for T-cells single live cell populations were gated based on CD5+ cells and expression of either CD4 (T-helper cells) or CD8 (cytotoxic T-cells), and B-cells were CD5− and CD21+. For analysis, fluorescence-minus-one controls were utilized to establish gating thresholds.

### 2.6. Systemic Immune Evaluation: Blood Processing

Given the scope of this manuscript, for all systemic immune evaluations, results are only reported for OS patients. Supplemental Figure S1 depicts a schematic of blood sample processing.

#### 2.6.1. Peripheral Blood Mononuclear Cell Immunophenotyping

Collected patient blood samples were utilized for immunophenotyping of circulating immune cell populations and assessment of monocyte function. Peripheral blood mononuclear cells (PBMCs) were isolated via density gradient separation with SepMate PBMC isolation tubes (Stem Cell Technologies, Vancouver, BC, Canada) following the manufacturer’s protocol. A portion of the PBMCs were stained with CD11b (clone M1/70), CD14 (clone M5E2), CD80 (clone 16-10A1), CD62L (clone FMC46) for monocyte phenotyping and CD5 (clone YKIX322.3), CD4 (clone YKIX302.9), CD8 (clone YCATE55.9), CD21 (clone LT21) and CD62L (clone FMC46) for lymphocyte phenotyping, and eFluor 506 fixable viability dye was used for viability assessment. The lymphocyte antibodies were purchased as described above, and CD11b, CD14, and CD80, were purchased from Biolegend (San Diego, CA, USA). Prior to staining with primary antibodies, the PBMCs were stained with canine-specific Fc inhibitor (polyclonal, Invitrogen, Waltham, MA, USA) following the manufacturer’s protocol. All primary antibody staining was conducted as follows: 1 × 10^6^ cells/100 µL were stained with the primary antibodies by incubating for 30 min at 4 °C in the dark, followed by a single wash step (cells pelleted via centrifugation at 300 *g* for 6 min) to remove unbound excess antibodies, and analyzed on CytoFLEX cytometer (Beckman Coulter, Brea, CA, USA). Flow data analysis was conducted with Kaluza software (V2.2, Beckman Coulter) based on the single live cell population. For monocytes, single live cells were further gated for positive expression of CD11b and CD14; the CD11b+ CD14+ population was gated for positive expression of CD80 or CD62L. For lymphocytes, T- and B-cells were gated as described in the tumor phenotyping section with the addition of the evaluation of positive expression of CD62L in the CD4 and CD8 T-cell populations. For analysis, fluorescence-minus-one controls were utilized to establish gating thresholds.

#### 2.6.2. Peripheral Monocyte Isolation

A portion of the isolated PBMCs (isolation described above) were utilized for monocyte sorting. Monocytes were sorted via positive selection for CD14+ cells via magnetic sorting with the MojoSort magnet (Biolgend, San Diego, CA, USA). For sorting, cells were resuspended at a concentration of 10–20 × 10^6^ cells/100 μL and incubated with canine-specific Fc inhibitor (Invitrogen), incubated with 1.25 µg of biotinylated anti-CD14 (clone M5E2) for 15 min on ice, followed by a wash step (centrifugation at 300 *g*, 6 min, at 4 °C), incubated with 2.5 µL of MojoSort streptavidin Nanobeads (Biolegend) for 15 min on ice, followed by a wash step, and then 5 magnetic sort steps following the manufacturer’s positive selection protocol (Biolegend).

#### 2.6.3. Peripheral Monocyte Cytokine Differential Gene Expression Analysis

Total RNA was extracted from a portion of the sorted monocytes with the DirectZol micro or mini prep RNA extraction kit (Zymo, Irvine, CA, USA) following the manufacturer’s protocol with the addition of a centrifugation step (10,000 *g* × 3 min) to pellet cellular debris after the addition of TriReagent. Total RNA quality has a 260/280 ratio of 1.8–2, and was then stored at −80 °C. The stored RNA was synthesized to cDNA with qScript cDNA SuperMix (QuantaBio, Beverly, MA, USA) used for assessment of *IFNg*, *IL10*, *TNF*, *GAPDH*, and *ACTB* gene expression via standard RT-qPCR with PerfeCTa SYBR Green, Low ROX (QuantaBio) on a 7500 Fast thermocycler (ThermoFisher). Primers were purchased from BioRad and sequences can be found in Supplemental Table S1. The standard ΔΔCt method was used for differential gene expression analysis.

#### 2.6.4. Peripheral Monocyte Functional Assessment

A portion of the sorted monocytes were then stimulated with lipopolysaccharide (LPS, *E. coli* 055:B5) in vitro at a concentration of 100 ng/1 × 10^6^ cells or left unstimulated as a negative control. The monocytes were cultured in DMEM supplemented with 10% FBS and 1% penicillin and streptomycin at 37 °C for 6 h. At 6 h, cell culture supernatant was collected and stored at −20 °C for future analysis. Monocytes were seeded at a density of 500,000 monocytes/mL in duplicates, and a total of ~1 mL of supernatant was collected, from pooled duplicate wells. This methodology was optimized in our laboratory to generate pilot data to support the use of 100 ng/1 × 10^6^ prior to use for clinical trial samples. Upon the completion of each study, groups’ samples were analyzed for chemokine and cytokines (IL10, IL8, MCP1 and TNFα) with a canine-specific Milliplex assay (Millipore Sigma, Burlington, MA, USA) and analyzed on a Luminex 200 (Luminex Corporation, Austin, TX, USA). Data was analyzed using the Belysa software (V1.2.1, Sigma Aldrich, St. Louis, MO, USA). The supernatant TGF-ß concentrations were assessed via ELISA (R&D technologies, North Kingstown, RI, USA) following the manufacturer protocol, and plates were read on a Syngergy HTX (BioTek, Winooski, VT, USA). For each assay a total of 25 or 50 μL of cell culture supernatant was utilized, respectively, with technical triplicates.

### 2.7. Statistical Analysis

Statistical analysis was conducted with GraphPad Prism Software (V 10.2.0) and significance cut off value was set to *p* < 0.05. For multiple groups and timepoint comparisons a two-way ANOVA was used for statistical analysis with Tukey post hoc test for multiple comparisons. For histology comparisons normality was determined by Shapiro–Wilk test, normally distributed data was analyzed with a one-way ANOVA Tukey post hoc test for multiple comparisons, and non-normally distributed data was analyzed with a non-parametric Kruskal–Wallis Test and post hoc Dunns test for multiple comparisons. For comparison of overall median survival time and disease-free interval outcomes, Kaplan–Meier curves were analyzed with a Mantel–Cox log-rank test. Survival time post-histotripsy was calculated based on the time elapsed from the histotripsy treatment date to the date of reported death/euthanasia. The disease-free interval (DFI) was calculated based on the time between limb amputation surgery and development of metastatic disease. Dogs with an unknown cause of death were presumed to have died from disease. The results from the MST stratification and MFI results for the patients that did not receive chemotherapy are underpowered and are interpreted as hypothesis-generating results. Further details on the Nanostring data and statistical analysis can be found in the appropriate methods section.

## 3. Results

### 3.1. Patient Demographics

A total of 32 dogs suspected to have OS were enrolled into the clinical trials and 28 dogs had a confirmed diagnosis of OS based on histology. A total of 10 patients received surgery 1 DPH, (1 DPH group) and 11 patients received surgery 3 DPH (3 DPH group), and 11 patients received surgery 5 DPH (5 DPH group) (Table S2). The average age and weight of the patients was 6.8 years (±2.5) and 38 kgs (±13.2) in the 1 DPH group, 6.5 years (±2.8) and 38.4 kgs (±17.8) in the 3 DPH group, and 9.1 years (±1.7) and 34.1 kgs (±11.7) in the 5 DPH group. Collectively, the average age of all enrolled dogs was 7.5 years (±2.6), the average weight was 37 kgs (±14), and the population was mixed-sex (Table S2). Of the 32 patients, demographics and bone tumor characteristics have previously been reported [20,25]: for patients 1–10 demographics were previously reported by Ruger et al. [20] and demographics for patients 19 and 26–32 were previously reported by Vickers et al. [25] (Table S2). A total of four patients were diagnosed with bone tumors other than osteosarcoma; two patients had chondrosarcoma, one hemangiosarcoma, and one osteoma.

### 3.2. Adverse Events

Histotripsy was successfully and safely delivered to all patients (n = 32) with no significant adverse events observed. Minor transient erythema and skin irritation post-histotripsy ablation was observed, but this resolved within 24 h post-delivery of histotripsy and has previously been reported and described in detail [20]. Anesthetic parameters monitored during histotripsy treatment stayed within normal ranges. These parameters included heart rate, respiratory rate, body temperature, systemic blood pressure, oxygen saturation levels, and cardiac rhythm via ECG. Patients did not exhibit any signs of adverse systemic signs after histotripsy ablation.

### 3.3. Histological Outcomes

**Figure 1:**
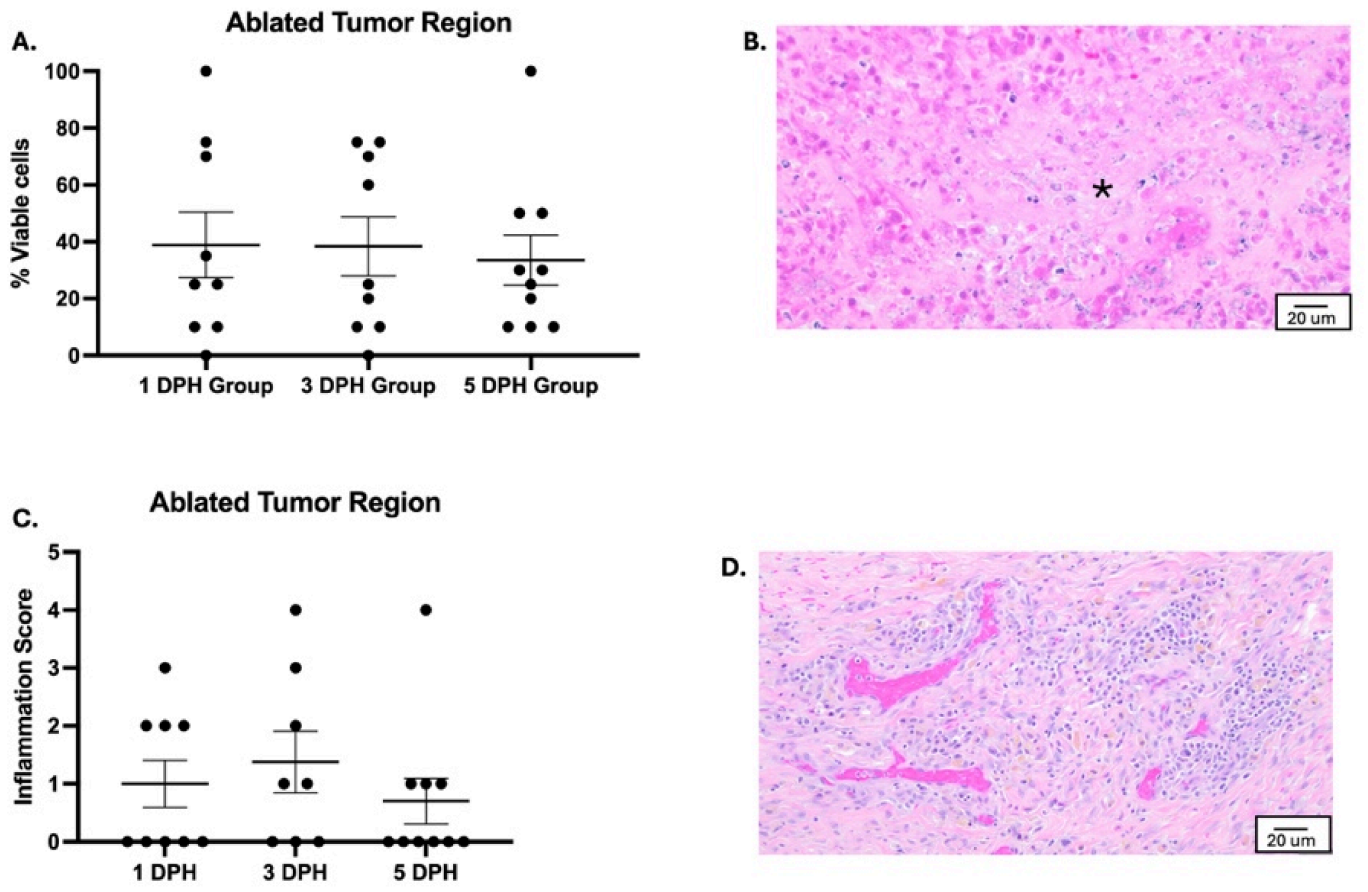
Histological assessment of cell viability and inflammation within histotripsy ablated regions of the tumor. (**A**) Percentage of viable cells in the examined tumor sections collected post-limb amputation surgery at 1, 3, or 5 DPH. (**B**) High magnification image from patient 11 illustrating the spectrum of morphologic changes indicating dead or dying cells. Within the center of the image (highlighted by the asterisk), individual cell borders are lost and replaced by fragments of nuclear debris (purple dots). The top left and bottom right of the image illustrate cell shrinkage, condensed cytoplasm, and loss of nuclear and cellular detail. (**C**) Scoring of inflammation within the histotripsy ablated tumor region. (**D**) High magnification image from patient 8 illustrating the finding of perivascular and periosteal lymphocytes, plasma cells, and macrophages, which was the predominant distribution and composition in samples where inflammation was present.

The microscopic and gross histological findings for patients 1–10 (Table S2) have previously been described [20] and previously been reported for patients 19 and 26–32 (Table S2) [25]. When comparing all patients’ tumor samples at the microscopic level (previously reported and those not previously reported), the percentage of viable cells and inflammation score of the histotripsy ablated tumor region did not differ significantly between groups (Figure 1A,C). Of the 32 tumor samples collected from patients enrolled in treat-and-resect histotripsy clinical trials, our group has not previously reported descriptive histological findings for 14 of these patients (patients 11–18, and 20–25, Table S2). In these 14 patients, the ablated tumor region displayed similar histological characteristics post-histotripsy ablation to those previously reported [20,25]. When present, mononuclear inflammation predominated and included macrophages, lymphocytes, and plasma cells while neutrophils were variable. Additionally, when present, inflammation was most consistently observed within the periosteum but was also observed cuffing vessels within the treated region or expanding regions of cell death (Figure 1B,D).

### 3.4. Immunomodulation of the Tumor Microenvironment (TME) Post-Histotripsy Ablation

**Figure 2:**
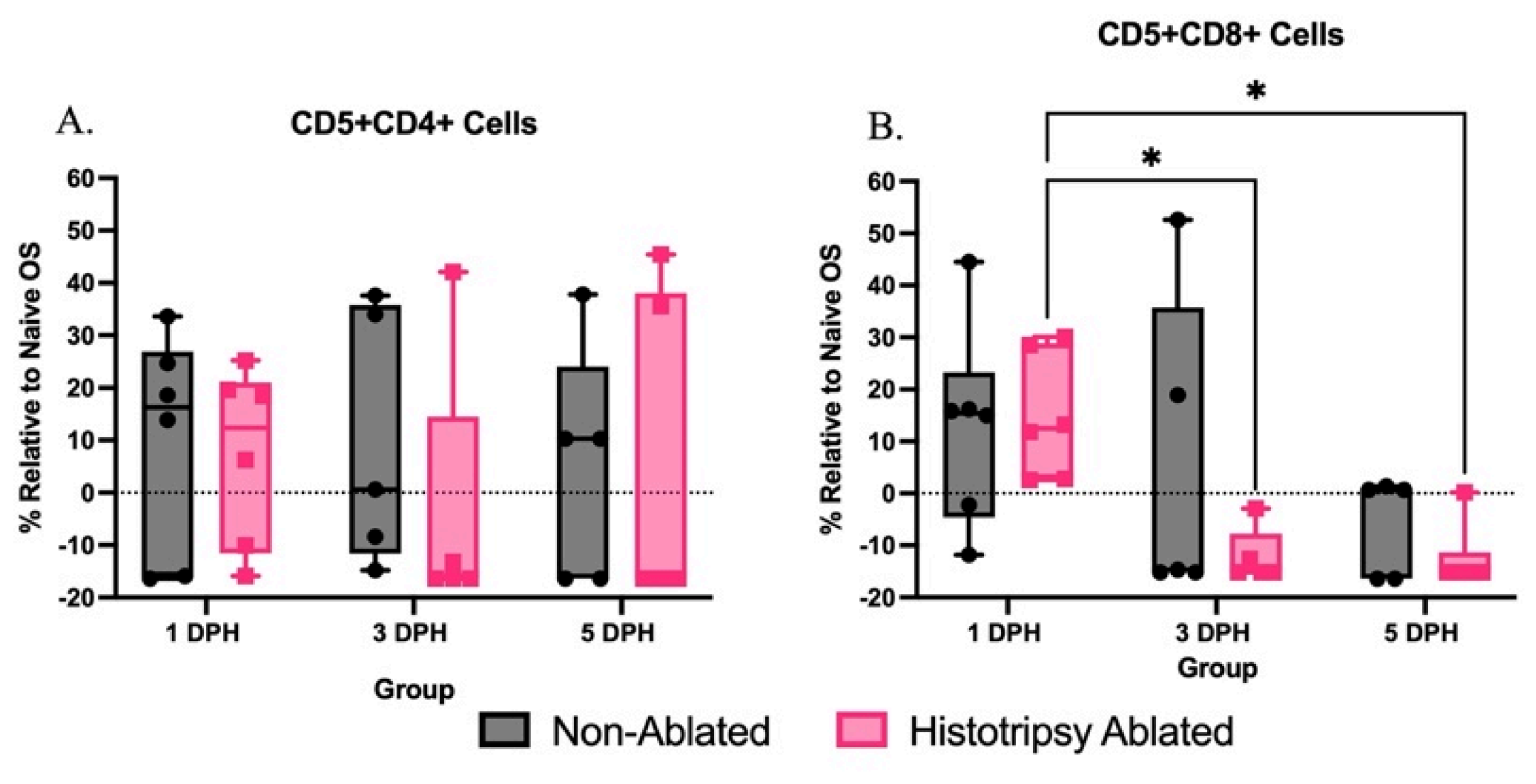
Evaluation of the immune tumor microenvironment. Paired histotripsy ablated and non-ablated tumor regions were collected from OS patients post-limb amputation surgery and (**A**) CD4 T-cells and (**B**) CD8 T-cell populations were evaluated relative to CD4, and CD8 T-cell populations found in naïve OS patient samples. Sample populations = 1 DPH Group (n = 6), 3 DPH Group (n = 5), and 5 DPH Group (n = 5 non-ablated and 6 ablated tumor regions). * *p* < 0.05. Two-way ANOVA with Sidak’s multiple comparison test.

T-cell populations of the non-ablated and ablated tumor regions were evaluated with flow cytometry. For all three groups the T-cell populations in patient paired non-ablated and ablated tumor regions are reported relative to the T-cell populations observed in treatment-naïve OS tumor samples. Overall, there were not any statistically significant changes in the CD4 T-cell populations amongst the three treatment groups. We observed changes in the median population of CD4 T-cells in ablated tumor regions, in the 3 and 5 DPH groups compared to the 1 DPH group (Figure 2A), and in the 3 and 5 DPH groups we observed changes in the median population of CD4 T-cells in non-ablated tumors compared to ablated tumors (Figure 2A). At 3 and 5 DPH there were minimal CD8 T-cells observed with statistically significant smaller populations of CD8 T-cells in histotripsy ablated tumor regions in these two groups compared to histotripsy ablated tumor regions at 1 DPH (Figure 2B). Overall, there was a change in the median prevalence of CD4 and CD8 T-cells in tumor samples collected 1 DPH in both the non-ablated and ablated tumor regions relative to naïve OS (Figure 2A,B).

### 3.5. Tumor Differential Gene Expression Analysis

**Figure 3:**
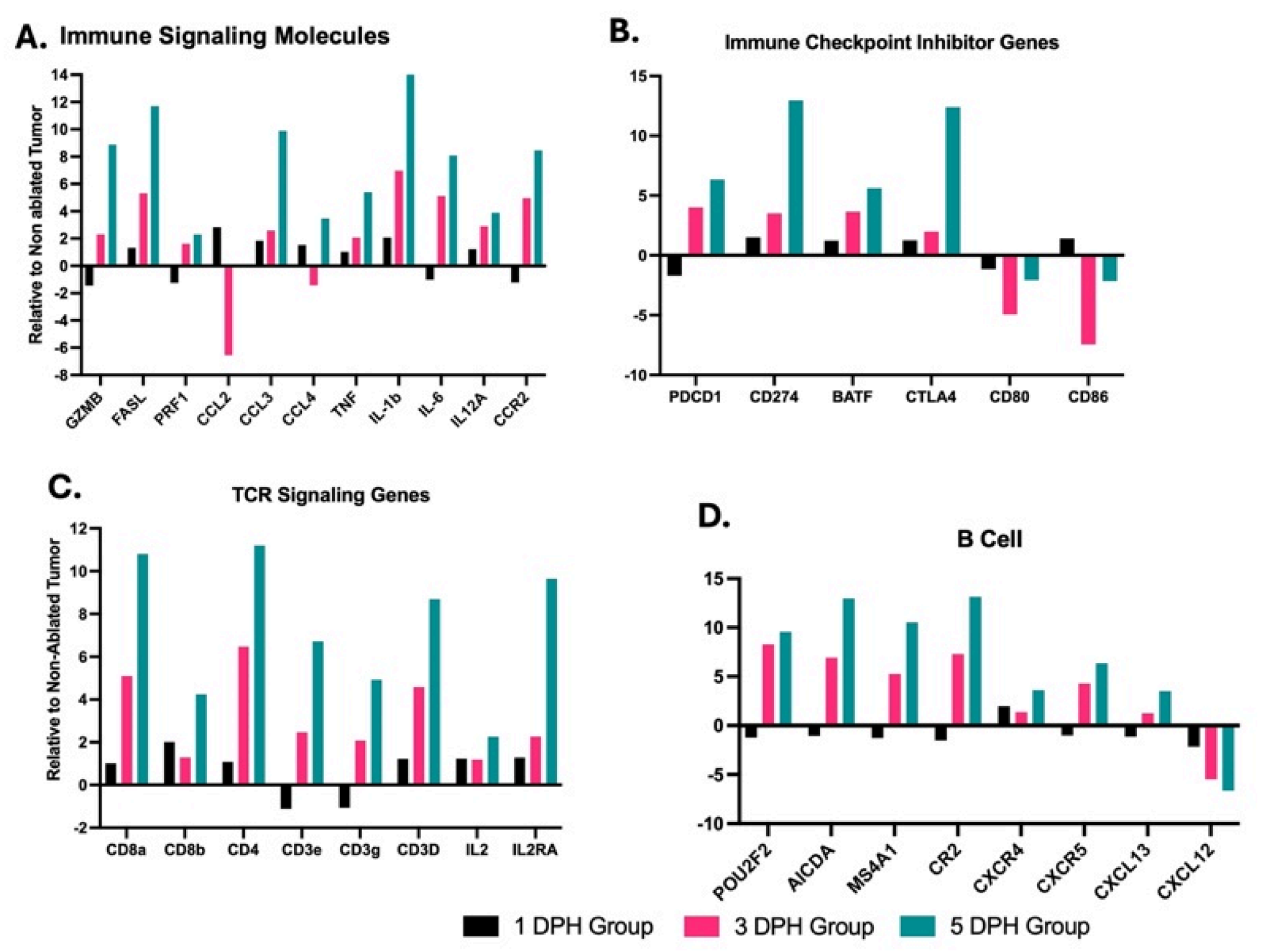
Evaluation of the effects of histotripsy on tumor immune gene signatures. Gene ontology determined relationships with the significantly altered genes. For (**A**–**D**) gene expression changes are being reported in ablated tumor regions relative to non-ablated regions for each of the three groups 1, 3, and 5 DPH. (**A**) Immune signaling molecules. (**B**) Immune checkpoint inhibitor. (**C**) TCR signaling pathway; (**D**) B-cell development and activation. n = 6 for each group. Refer to Methods and Results sections for further details regarding statistical analyses which were conducted in nSolver (Bruker, Seattle, WA, USA). *p* values presented in Supplemental Table S3.

To dive deeper into the local immunomodulation induced by histotripsy we conducted differential gene expression analysis using the Nanostring Canine Immuno-Oncology panel. The most robust gene expression changes were observed in tumor samples collected from the 5 DPH group. Histotripsy ablated tumor regions revealed an upregulation of inflammatory cytokines and chemokines (*CCL3*, *CCL4*, *TNF*, *IL1β*, *IL6*, *IL12A*) and cytotoxicity genes (*GZMB*, *FASL*, *PRF1*) (Figure 3A) relative to non-ablated tumor regions. An area of interest in the field of focused ultrasound is pairing focused ultrasound modalities with immunotherapies such as immune checkpoint inhibitors (ICIs) to improve the efficacy of ICIs for solid tumors [36,37]. Therefore, the PD1, CTLA4, and TCR signaling pathway gene signatures were evaluated in collected tumor samples to assess histotripsy associated changes. There was a significant upregulation of ICI genes *PDCD1* (6.35), *CD274* (12.96), *BATF* (5.62), *CTLA4* (12.4) and an insignificant downregulation of CTLA4 ligands *CD80* (−2.06) and *CD86* (−2.14) (Figure 3B and Table S3). There was a significant upregulation (*p* < 0.05) of TCR genes *CD3e* (6.72), *CD3g* (4.93), *CD3d* (8.69), co-receptor genes *CD8a* (10.8), *CD8b* (4.24), *CD4* (11.2), co-stimulators *CD28* (8.93), *ICOS* (10.2), and *CD40L* (8.4) and downstream activation genes *IL2* (2.26) and *IL2RA* (9.64) in histotripsy ablated tumor regions compared to non-ablated tumor regions (Figure 3C and Table S3). Additionally, we characterized B-cell signatures and observed an upregulation of B-cell activation genes including *POU2F2* (9.56), *AICDA* (12.96), *MS4A1*(10.53), *CR2* (13.14), *CXCR4* (3.62), *CXCR5* (6.35), and *CXCL13* (3.5) (Figure 3D, Table S3). While we observed the greatest differential gene expression changes in the 5 DPH group, there were also changes in the aforementioned gene signatures in the 1 and 3 DPH groups, with the changes in the 1 DPH group being the least (Figure 3, Table S3).

Additionally, non-ablated and ablated tumor gene signatures relative to treatment-naïve OS tumor samples were evaluated (Figure S2 and Table S4). These changes were not statistically significant, but a greater upregulation of the genes mentioned above was observed in the histotripsy ablated tumor regions compared to treatment-naïve sections, primarily at 5 DPH. In general, we observed opposing expression patterns in the non-ablated tumor regions compared to ablated regions (Figure S2 and Table S4). One additional interesting finding was the observation of the upregulation of TCR signaling and anti-PD1 signaling genes within the non-ablated tumor region in the 3 DPH group (Figure S2B).

### 3.6. Systemic Immune Evaluation: Peripheral Monocyte Evaluation

**Figure 4:**
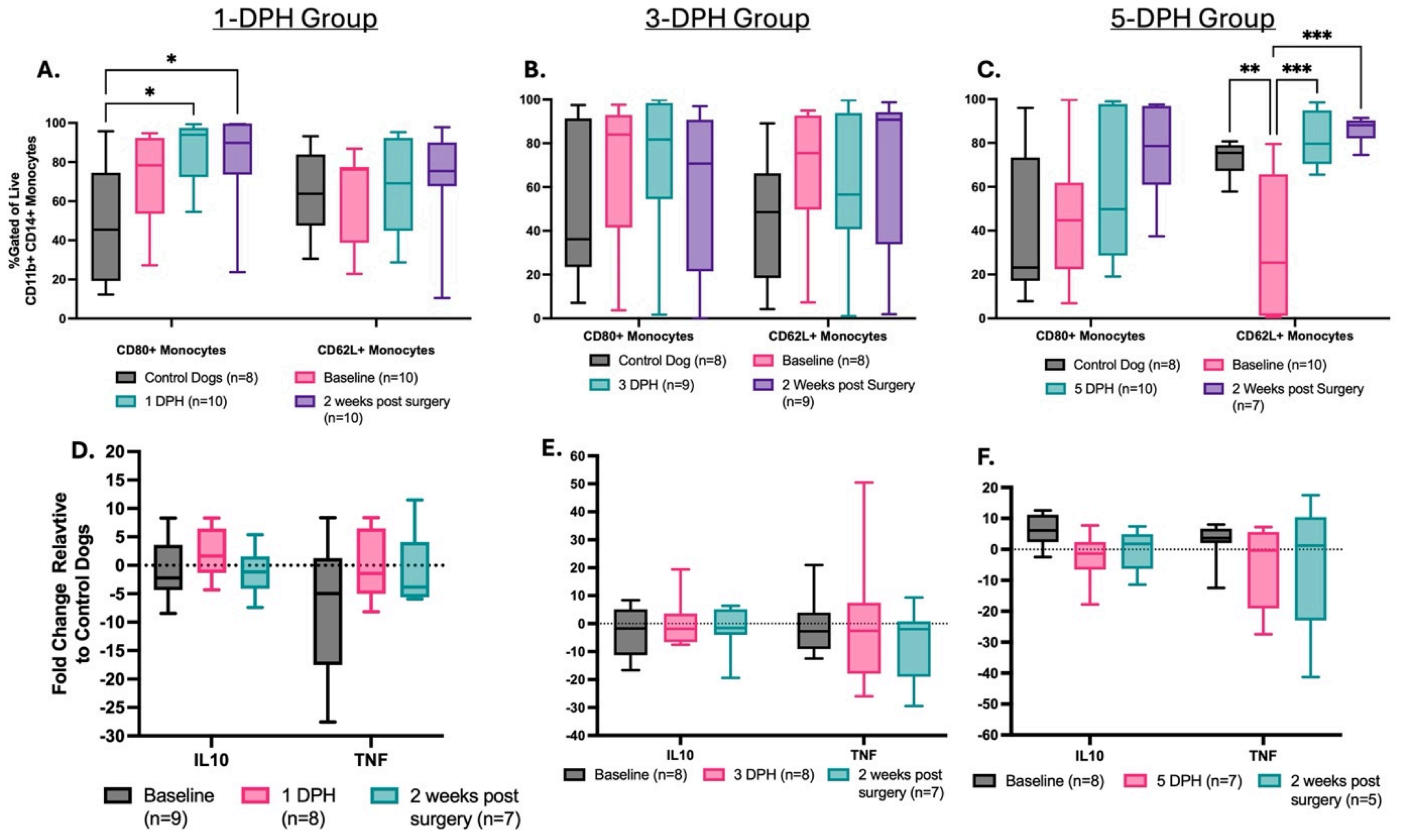
Characterization of peripheral monocyte phenotypes post-histotripsy ablation. CD80 and CD62L monocyte expression was characterized in the (**A**) 1 DPH group, (**B**) 3 DPH group, and (**C**) 5 DPH group, and the monocyte gene expressions for *IL-10* and *TNF* were evaluated for the (**D**) 1 DPH group, (**E**) 3 DPH group, and (**F**) 5 DPH group. * *p* < 0.05, ** *p* < 0.005, and *** *p* ≤ 0.0005. Two-way ANOVA with Tukey test for multiple comparisons.

In the 1 DPH group there was a statistically significant increase in CD80-expressing monocytes at 1 DPH (*p* = 0.01) and 2 weeks post-surgery (*p* = 0.02) compared to healthy control dogs (Figure 4A). In the 3 DPH group there were no statistically significant changes in CD80 and CD62L within the monocyte population. In the 3 DPH group we observed no significant changes in monocyte CD80 and CD62L changes (Figure 4B). In the 5 DPH group we observed a statistically significant increase in monocyte CD62L expression at 5 DPH (*p* = 0.0003), and 2 weeks post-surgery (*p* = 0.0005) compared to baseline and significantly less monocytes at baseline compared to healthy control dogs (*p* = 0.005) (Figure 4C).

The expression of the inflammatory genes *IL10*, *TNF*, and *IFNG* were assessed in monocytes isolated from each OS patient. In the 1 DPH group, at 1 DPH there was an insignificant upregulation of *IL10*, and *TNF* compared to baseline and gene expression declined by 2 weeks post-surgery (Figure 4D). Similar to the immunophenotype findings there were minimal changes in the assessed gene expression patterns in the 3 DPH group (Figure 4E). In the 5 DPH group there was an insignificant downregulation of *IL10* and *TNF* at 5 DPH and 2 weeks post-surgery compared to baseline, with a 7-fold and 4-fold downregulation of *IL10* and *TNF*, respectively (Figure 4F). In all assessed monocytes limited to no detectable expression of *IFNG* was observed.

**Figure 5:**
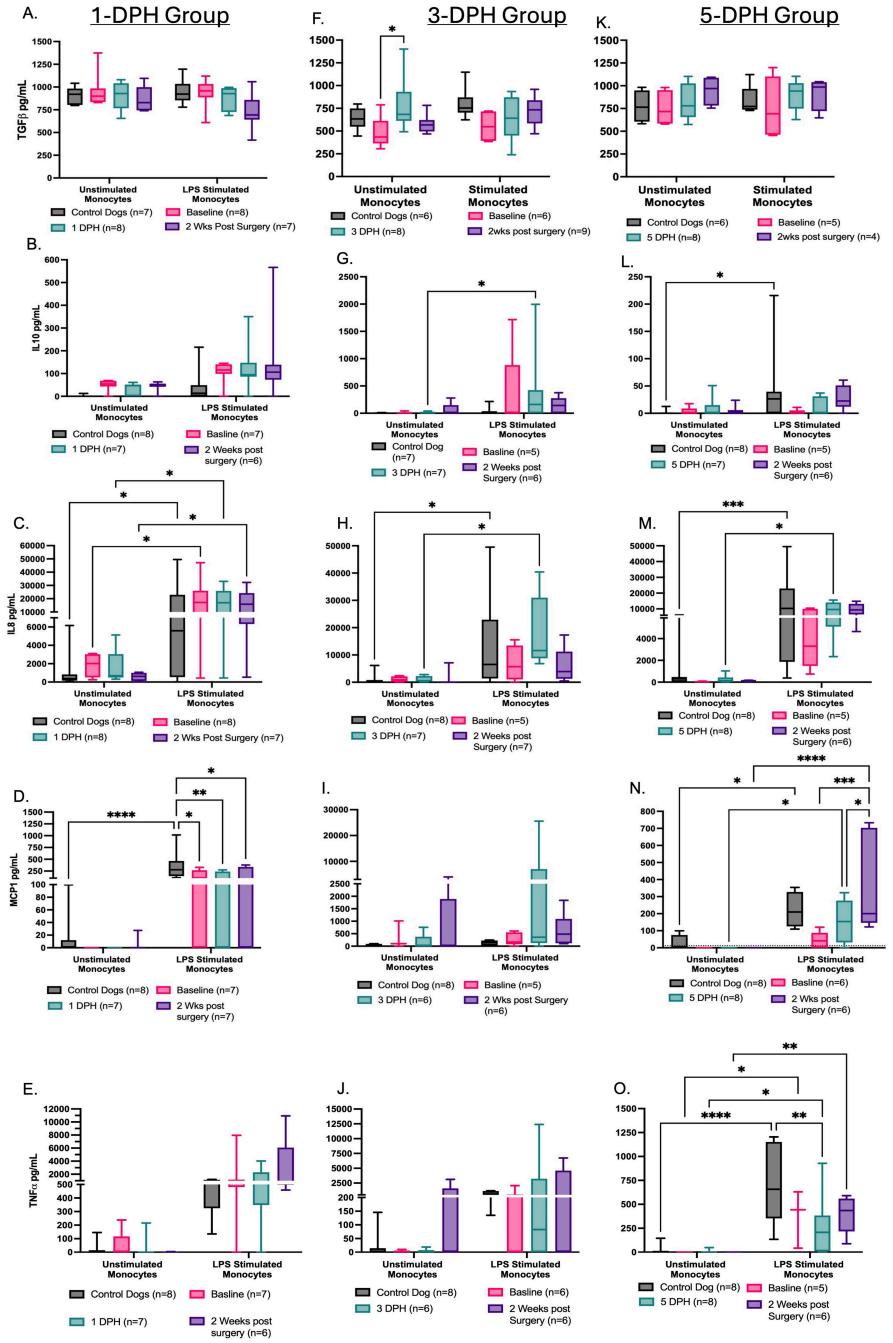
Functional assessment of patient isolated monocytes. Patient monocyte response to lipopolysaccharide (LPS) was assessed via cytokine and chemokine production. Assessed cytokines and chemokines included TGFβ, IL-10, IL8, MCP-1, and TNFα. (**A**–**E**) Results from the 1 DPH group, (**F**–**J**) results from the 3 DPH group, and (**K**–**O**) results from the 5 DPH group. * *p* < 0.05, ** *p* < 0.005, *** *p* ≤ 0.0005 and **** *p* ≤ 0.0001. Two ANOVA with Tukey test for multiple comparisons.

Lastly, post-in vitro stimulation with LPS, production of inflammatory cytokines and chemokines was assessed in patient-isolated monocytes. Overall, the monocytes isolated from dogs in the 1 DPH group behaved similarly across all time points with respect to chemokine and cytokine production post 6 h of stimulation with LPS, and responses closely paralleled those of control dogs (Figure 5A–E). Post-activation, the monocytes isolated from dogs in the 3 DPH group had a cytokine and chemokine profile indicative of histotripsy induced immunomodulation with a significant increase in IL8 (*p* = 0.01) and IL10 (*p* = 0.03) production at 3 DPH in stimulated monocytes compared to unstimulated monocytes (Figure 5E–H), and unstimulated monocytes produced significantly greater TGFβ (*p* = 0.03) compared to baseline production levels (Figure 5F). In the monocytes isolated from dogs in the 5 DPH group the histotripsy induced inflammatory response was more robust with IL8 (*p* = 0.03), MCP-1 (*p* = 0.01), and TNFα (*p* = 0.03) significantly increased at 5 DPH in stimulated monocytes compared to unstimulated one. Furthermore, in this group TNFα (*p* = 0.008) and MCP-1 (*p* < 0.0001) continued to be significantly increased in stimulated monocytes compared to unstimulated 2 weeks post-surgery, and across all timepoints TGFβ and IL10 remained relatively unchanged (Figure 5K–O).

### 3.7. Systemic Immune Evaluation: Lymphocyte Phenotyping

**Figure 6:**
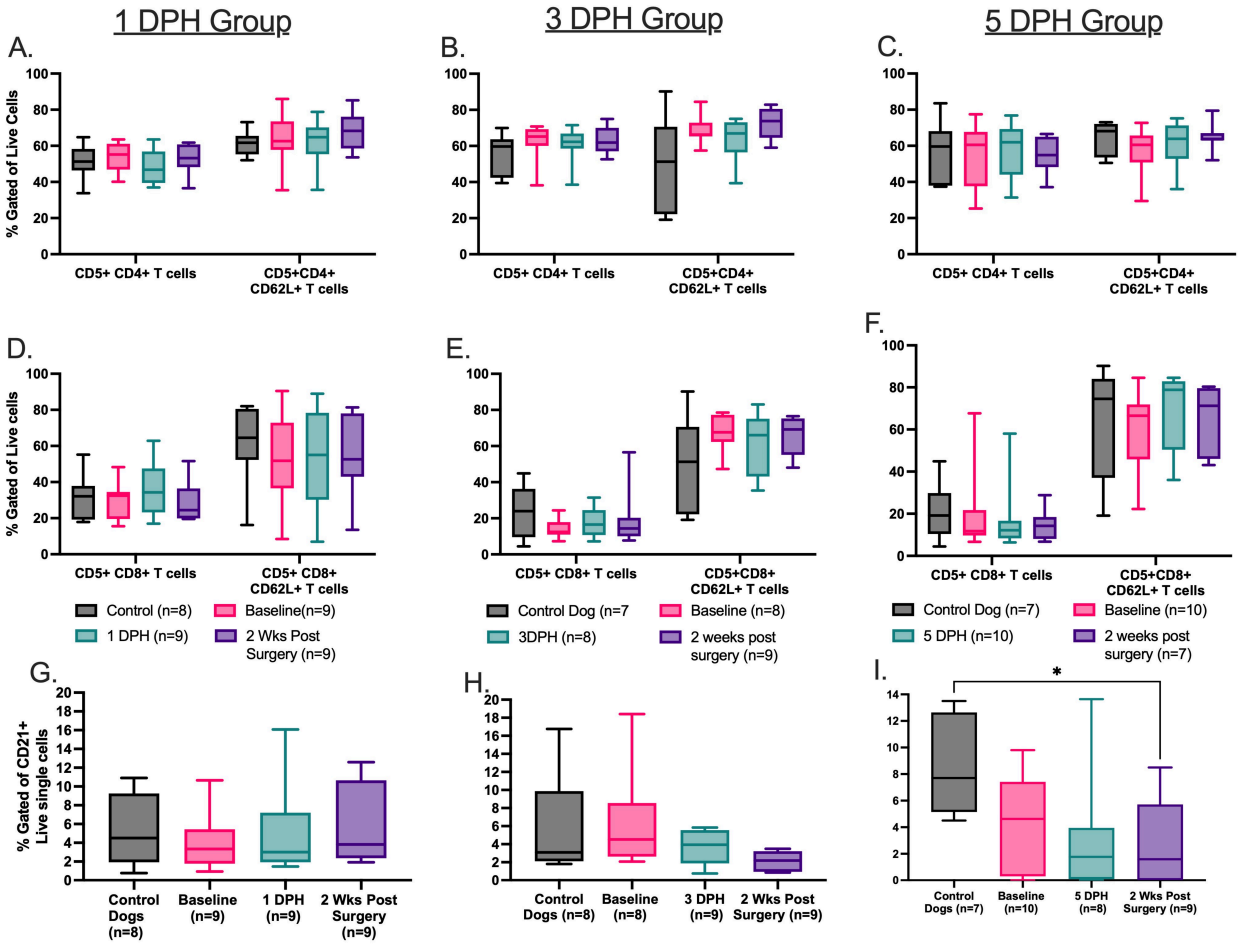
Characterization of peripheral lymphocytes post-histotripsy ablation. T and B-lymphocyte populations were assessed via flow cytometry in (**A**–**C**) the 1 DPH group, (**D**–**F**) the 3 DPH group, and (**G**–**I**) the 5 DPH group. * *p* < 0.05.

In all groups we did not observe any statistically significant changes in circulating T helper (CD4) or cytotoxic (CD8) T-cell populations or expression of adhesion molecule CD62L within the T-cell populations compared to baseline Figure 6A–F. Similar to the T-cell population observations there were minimal changes in the circulating B-cell (CD21) populations (Figure 6G–I). In the 5 DPH group there was a smaller B-cell population in OS patients (3%) compared to age- and weight-matched healthy control dogs (9%); this difference in B-cell populations was statistically significant at 2 weeks post-surgery (*p* = 0.02) (Figure 6I).

### 3.8. Osteosarcoma Patient Clinical Outcomes

**Figure 7:**
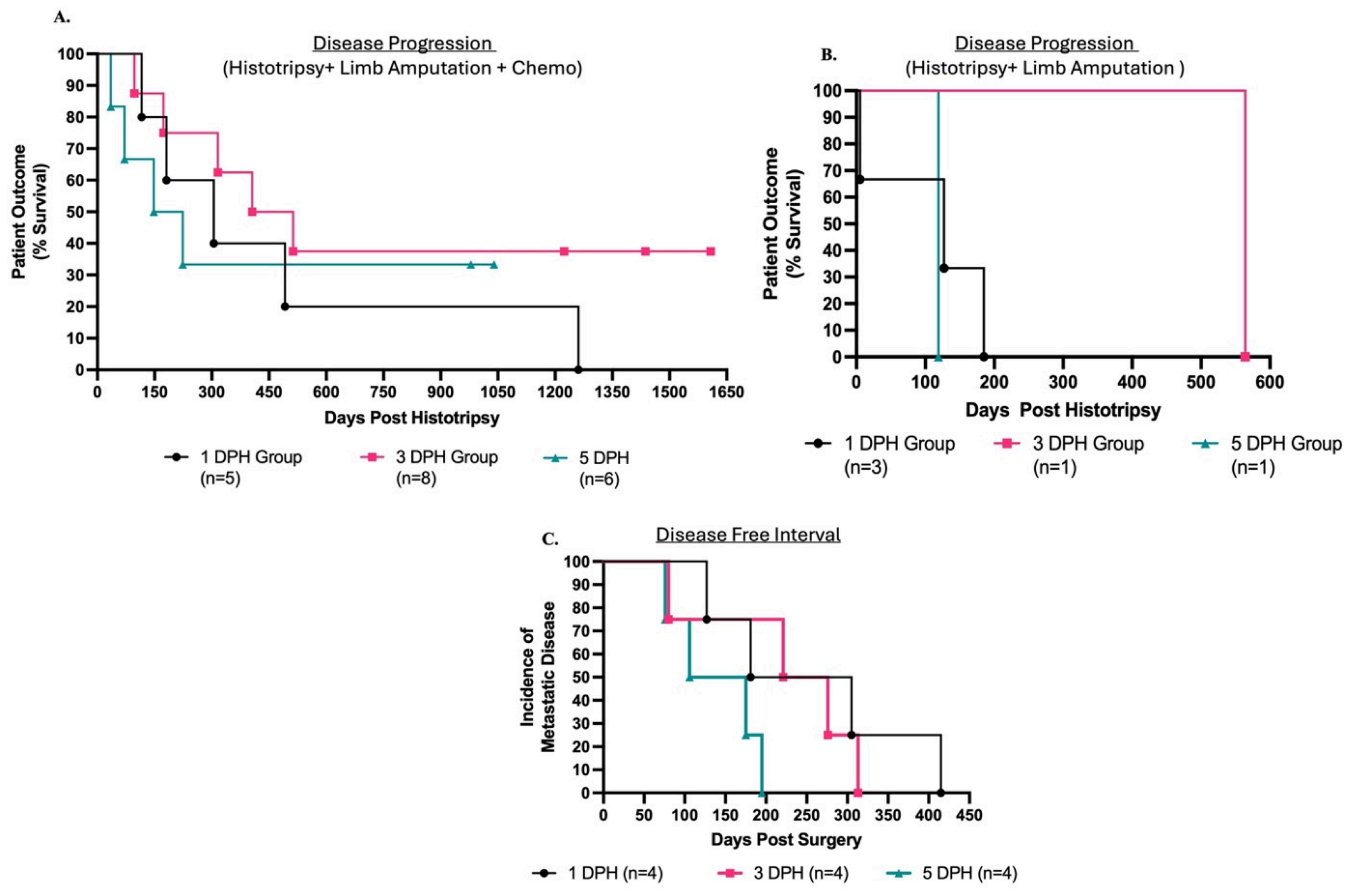
Disease progression evaluation in osteosarcoma patients. Disease progression -post-delivery of histotripsy in each of the three groups (1, 3, and 5 DPH). (**A**) OS patients that received definitive standard-of-care (limb amputation surgery + adjuvant chemotherapy) and histotripsy and (**B**) patients who received histotripsy and limb amputation surgery only. (**C**) Disease-free interval (time between limb amputation surgery and development of metastatic disease) was determined for the patients that received histotripsy + definitive standard-of-care. No statistically significant differences between groups were observed. DPH = Days post-histotripsy.

A total of 24 of the 28 OS patients were followed until end of life or were alive at the time of writing this manuscript, and four OS patients were lost to follow-up. Of the 24 patients with follow-up data, 19 received adjuvant chemotherapy (carboplatin or doxorubicin as part of the definitive standard of care) post-limb amputation surgery following a standard-of-care treatment protocol. Of these 19 patients that received chemotherapy, the median survival time (MST) was 305 days (97–1261 days) in the 1 DPH group (n = 5), 459.5 days (97–1420 days) for the 3 DPH group (n = 8), and 186 days (36–1040 days) for the 5 DPH group (n = 6). The collective MST was 316 days (36–1608 days) (Table 1). The 3 DPH group had the longest MST (Figure 7A). When considering that the expected MST for canine OS patients who receives definitive standard-of-care (limb amputation surgery + adjuvant chemotherapy) is 10–12 months [38,39,40], nine patients (34.1 months MST) surpassed the expected reported MST (Figure 7A). Of these nine patients (47% of patients with follow-up data), three patients survived for >1 year post-histotripsy and six patients survived for >2 years (Table 1, Figure 7A). Of the 24 patients with follow-up data, five did not receive chemotherapy (owner elected) and the collective MST was 127 days (5–564 days); three patients were in the 1 DPH group, and one each were in the 3 or 5 DPH groups (Figure 7B, Table 1).

Of the 19 OS patients with follow-up data that received histotripsy + definitive standard of the date, metastatic disease was first observed (via thoracic radiographs) and was known for 12 of these patients. Of these 12 patients collectively, the median DFI was 184 days (76–415 days), and the median DFI for dogs in the 1 DPH group (n = 4) was 243 days, in the 3 DPH group (n = 4) 248.5 days, and in the 5 DPH group (n = 4) 140.5 days. There were no statistically significant differences between the three groups (Figure 7C, Table 1).

All OS patients with follow-up data that received histotripsy + SOC were further stratified based on survival time into three categories: expected MST (MST 10–12 months); below MST (<10 months); and above MST (>12 months). In an exploratory manner with the goal of generating hypothesis-generating results, we evaluated factors that may have influenced disease progression. We found minimal differences between circulating T- or B-lymphocyte or monocyte populations. There was a modestly greater population of T-cells in the below and above MST groups compared to the expected MST group (Figure S3A). There was a greater population of B-lymphocytes in the above MST group compared to the below and expected MST groups, observed at baseline and day of surgery (Figure S3B). A greater population of monocytes was observed at baseline in the below and above MST groups compared to the expected MST group (Figure S3C). We did not observe differences in the evaluated histological parameters of inflammation score and % viable cells in treated tumor region (Supplemental Figure S3D,E).

## 4. Discussion

This long-term study reports on the immunological and clinical outcomes of 28 canine OS patients enrolled in safety and feasibility clinical trials for assessment of histotripsy ablation and is the first larger-scale investigation of outcomes associated with histotripsy ablation for OS. We demonstrated that partial tumor volume ablation with histotripsy prior to definitive standard-of-care can potentially induce immunomodulation and patient survival, and disease-free outcomes in this study were comparable to previously reported median survival data [38,39,40] and, in some cases, exceeded these expected medians. Our primary focus of the study was canine patients with OS, due to the prevalence of OS in dogs and the high degree of translational relevance between canine and human OS patients [5,7]. Thus, immunological outcomes were only reported for the 28 canine OS patients in this study. Extensive evaluation of the ablative outcomes was not within the scope of this manuscript as this has previously been reported for 18 of the 32 patients mentioned in this study [20,25]. We observed that the safety and histologic outcomes for the previously unreported 14 patients in this study are similar to the 18 previously reported patients. Briefly, the major finding of ablative outcomes of the total patient population presented in this manuscript was the successful identification of gross and microscopic histotripsy ablation zones within the targeted tumor region, and planned ablated volumes closely matched what was observed on gross histology. Microscopic evaluation of ablation zones demonstrated similarly extensive foci of hemorrhage and necrosis, coagulative and lytic cell death, and varying degrees of matrix degeneration [20,25]. Including the 14 previously unreported patients, we now show that histotripsy tumor ablation was safe and well tolerated by all 32 patients.

Understanding the immunomodulatory capacity of histotripsy ablation is essential for advancing histotripsy for clinical application. The high value of immunomodulatory potential is due to the need to develop innovative treatment options which alter the immunosuppressive TME in order to mitigate metastatic disease. In the context of systemic immunomodulation, we observed several promising results which warrant further investigation. These results include a transient effect of histotripsy on CD80 monocyte expression with only a statistically significant increase observed at 1 DPH and 2 weeks post-surgery compared to healthy control dogs in the 1 DPH group. In the 1 DPH group the increased CD80 expression did persist for up to two weeks post-surgery, and this trend was observed in the 5 DPH group as well, but the influence of surgery should be considered at this timepoint. Additionally, within the 5 DPH group we observed a statistically significantly greater population of CD62L expressing monocytes 5 DPH and 2 weeks post-surgery, suggesting increased monocyte chemotaxis (Figure 4A–C). Our in vitro LPS monocyte stimulation results further support the increased activation state of monocytes in the 5 DPH group with greater levels of TNFα, IL-8, and MCP-1 (CCL2) in LPS stimulated monocytes compared to unstimulated monocytes post-histotripsy (Figure 5K–O). In an in vivo study, the migration of monocytes via the CCL2-CCR2 axis has previously been reported to be potentially linked to metastasis in canine OS [41]. While we did not observe any unexpected metastases patterns, this finding is still worth acknowledging and continued investigation is warranted to determine the clinical relevance of our in vitro results. Conversely, in cell culture setting the dysregulation and impaired chemotaxis of circulating monocytes in canine OS patients [42] and the importance of monocytes in OS anti-tumor immunity have previously been reported on [43]. Furthermore, we observed similar populations of monocytes (based on flow cytometry) in both the below and above MST groups of dogs in the current study, further highlighting the dynamic role monocytes likely play in OS patient outcome (Figure S3C).

Somewhat expectedly, we observed minimal changes in our circulating T- and B-cell populations post-delivery of histotripsy. This is likely due to the sampling timepoints selected; as adaptive immune cells, T- and B-cell activity peaks closer to 7–14 days post-immunomodulatory event. However, we did observe a decreased B-cell population at 2 weeks post-surgery in the 3 and 5 DPH groups compared to baseline and the healthy control dogs (statistically significant in the 5 DPH group compared to healthy control dogs) suggesting an influence of histotripsy on B-cell populations. This was further supported by our observation of greater circulating B-cell populations and improved patient outcomes (Figure S3B). Furthermore, locally within histotripsy ablated tumor regions, we observed an upregulation of genes associated with B-cell activity and migration in the 3 and 5 DPH groups (Figure 3D). The potential link between the observed local and systemic B-cell population changes proposes an exciting area of future investigation. The role of B-cells in anti-tumor immunity is not fully elucidated, especially in the context of OS. However, anti-tumor B-cells are uniquely poised to influence both innate and adaptative anti-tumor immunity, making their role in the TME and patient prognosis an increasing area of interest in the field of immuno-oncology [44].

Furthermore, locally within the histotripsy ablated tumor regions, we observed significantly less cytotoxic (CD8) T-cells in tumors collected 3 and 5 DPH compared to 1 DPH, suggesting that the presence of cytotoxic T-cells is potentially limited at 3 or 5 DPH. This limited T-cell population might be a result of sampling time point or poor cell viability within the ablated tumor region, which could be expected. However, we did observe the upregulation of essential anti-tumor immune signaling molecules associated with a pro-inflammatory cytotoxic T-cell response, including *CD8a*, *CD8b*, *GZMB*, *FASL*, *TNF* and *IL12A* (Figure 3A,C). These gene signature patterns suggest that a T-cell response was initiated by histotripsy ablation, potentially becoming more robust over time. However, they do not indicate a tumor antigen-specific immune response and further TCR sequencing analyses are needed to draw specific anti-tumor T-cell response conclusions. The differences between the flow cytometry immunophenotyping and Nanostring gene signature results can be attributed to the difference in assay type (protein assessment vs. gene expression assessment), or sampling; flow cytometry and Nanostring tumor samples were adjacent samples, but the assays were not conducted with the same exact sample.

Our gene ontology results indicated the upregulation of common ICI genes and signaling pathways, including *PD1* and *CTLA4*, in histotripsy ablated tumor regions relative to non-ablated tumor regions (Figure 3B). This is especially important because as the field of histotripsy advances, combination treatment strategies of focused ultrasound + ICIs are being investigated with the hypothesis that focused ultrasound ablation modalities such as histotripsy improve the efficacy of immunotherapies [36,37]. The efficacy of ICIs in solid tumors, including OS, is poor. However, it has been previously reported that in OS, PD1 and PDL1/L2 expression is associated with disease progression and development of metastatic disease [45,46], and immunotherapy has been reported to decrease metastatic disease in preclinical rodent models [47]. The safety of anti-PD-1 as a monotherapy for human OS has been established, but the efficacy is very limited, likely due to the inherent immunosuppressive TME [47,48]. Additionally, anti-CTLA4 has been reported to inconsistently increase overall survival in a limited population of pediatric OS patients, suggesting poor efficacy of anti-CTLA4 as a monotherapy [49]. In preclinical rodent models, the use of focused ultrasound ablation and ICIs demonstrates promising synergistic effects, evident from the improved efficacy of the ICIs [30,37,50,51]. Together these results support the potential for improved efficacy of ICIs for OS if combined with histotripsy and future studies investigating the combination therapies, especially warranted in canine patients with biologically relevant spontaneously occurring disease.

The reported expected MST for canine OS patients that received definitive SOC is 10–12 months [38,39,40]. Collectively, the MST for the 19 patients that received histotripsy + SOC was 10.4 months, aligning with the expected reported MST. Excitingly, a total of five patients that received histotripsy + SOC were alive and doing well at the time of writing this manuscript, with a median progression-free survival time of 3.4 years post-delivery of histotripsy. There are limited studies reporting on 3+ year survival time of OS patient populations that received SOC, presumably due to the rarity of this outcome. One previous study reported 1 of 14 dogs (7%) survived 3 years with SOC [52]. For 2+ year survival previous studies have reported 9.7% of a study population of 35 dogs [53], and 24.8% 2-year survival of dogs in a study population of 324 dogs [54]. The 26% 3+ year median survival in the current study observed with histotripsy + SOC warrants further investigation with studies with larger populations, more complete histotripsy ablation, and combination therapies, to determine the influence of histotripsy on patient survival. Furthermore, it has previously been documented that the MST for canine OS patients receiving tumor-bearing limb amputation surgery only and no adjuvant chemotherapy is 134.4 days with 11% and 2% of dogs surviving 1 and 2 years, respectively [55]. In the current study, for the dogs that received histotripsy + limb amputation surgery only, the MST was 127 days, with one dog (20%) surviving for 1.5 years.

To provide further insight into patient outcomes we evaluated circulating monocyte and lymphocyte populations, as it has previously been reported that complete blood cell counts from OS patients demonstrated high monocyte and lymphocyte counts and were associated with poorer prognosis in canine OS patients [56]. Interestingly, we did observe a greater population of circulating B-lymphocytes (cells) in the above median responders (MST > 12 months) group compared to the expected (10–12-month MST) and below median responders (MST < 10 months) at baseline and day of surgery, suggesting a potential positive correlation between B-cells and patient outcomes (Figure S3B). We also evaluated the differences in cell viability and inflammation of histotripsy ablated tumor regions and hypothesized that improved patient outcome would be associated with improved histotripsy ablation (i.e., higher percentage of non-viable cells and greater amount of inflammation). However, we observed no differences between these histological parameters and patient outcome (Figure S3D,E). We did not observe statistically significant differences in MST between the three study groups (1, 3, or 5 DPH) suggesting that the time of limb amputation surgery post-histotripsy may not significantly influence patient disease progresion in this study. Similar to human OS, canine OS is incredibly heterogenous, which likely contributes to the differences in patient responses to treatment. Gaining further insight and understanding of patient outcomes post-histotripsy ablation is essential for clinical advancement of histotripsy as a mono- or combination therapy for OS, and continued investigations are warranted.

As our team continues to further develop histotripsy ablation for OS, we are working towards developing devices to efficiently achieve larger ablation volumes and customized ablations to more effectively target the intratumoral heterogeneity that is commonly observed in canine OS tumors. Osteosarcoma is often composed of differing proportions of bone and soft tissue, with the hardness of bony regions varying from lytic bone to intact cortical bone. Device advancements that address the variation in tumor tissue composition are needed to improve ablation efficiency and efficacy and provide patient tumor-specific treatment plans. As we work towards advancing clinical application of histotripsy for OS, future investigations will include optimizing dose, as recent work in the focused ultrasound field suggests that there is an optimal immunomodulatory histotripsy dose [57,58]. Identifying this dose will be especially important for future studies which aim to combine histotripsy with immunotherapies such as ICIs. Combination histotripsy therapies will be essential for further boosting the anti-tumor immune response and mitigating primary disease recurrence (in a treat-and-leave setting) and metastatic disease.

## 5. Conclusions

We demonstrated that histotripsy may induce both local and systemic immunomodulatory effects in spontaneously occurring canine OS. At 5 DPH, circulating monocyte activation was evident. Furthermore, gene signatures of ablated tumor regions indicated the upregulation of TCR signaling, inflammatory signaling pathways, and immune checkpoint molecules, specifically *PD1*, *CTLA4*, and *PD1L* relative to non-ablated tumor regions. The upregulation of immune checkpoint genes supports the motivation for future studies which aim to combine immune checkpoint inhibitors (ICIs) with tumor ablation to improve the efficacy of these immunotherapies with the ultimate goal of improving patient prognosis. Overall, the results in this manuscript highlight the immunomodulatory potential of histotripsy and these hypothesis-generating results serve as the foundation for future work which aims to further advance the immunomodulatory capabilities of histotripsy.

## Figures and Tables

**Table 1:** Patient outcomes. Patient # corresponds to patient # in Table S2. Surgery group is number of days post-histotripsy that limb amputation surgery occurred. *Reason of Death* = *unknown:* patient death was reported by owner without reported cause; the cause of death is presumed to be related to OS.

Patient #	Surgery Group	Survival		Days Post-Surgery till Mets (DFI)	Days Post-Completion of Chemo till Mets	Days Post-Mets till Death	Reason for Death
		Patient Status	Days Post-Histotripsy				
1	1	Euthanized	181	181	Unknown	Unknown	Pulmonary Metastatic Disease
2	1	Euthanized	492	415	269	223	Pulmonary Metastatic Disease
3	1	Euthanized	185	184	No Chemo	1	Pulmonary Metastatic Disease
4	1	Euthanized	1261	No Mets	No Mets	No Mets	Injury
5	1	Died	116		No Mets	No Mets	Unknown
6	1	Euthanized	305	305	Unknown	Unknown	Pulmonary Metastatic Disease
7	1	Euthanized	127	95	No Chemo	32	Pulmonary Metastatic Disease
8	1	Euthanized	5	No Mets	No Chemo	No Mets	Unknown
11	3	Alive	1608	No Mets	No Mets	N/A	N/A
12	3	Alive	1437	No Mets	No Mets	N/A	N/A
14	3	Alive	1224	No Mets	No Mets	N/A	N/A
15	3	Euthanized	97	80	0	17	Pulmonary Metastatic Disease
16	3	Euthanized	564	Unknown	No Chemo	Unknown	Unknown cause
17	3	Euthanized	316	221	69	95	Pulmonary Metastatic Disease
18	3	Euthanized	513	276	154	237	Pulmonary Metastatic Disease
19	3	Euthanized	173	158	0	15	Bone Metastases
21	3	Euthanized	406	313	167	93	Pulmonary Metastatic Disease
24	5	Euthanized	148	106	21	42	Pulmonary Metastatic Disease
22	5	Euthanized	71	No Mets	0	N/A	Chemo complications
26	5	Alive	1040	175	84	N/A	
27	5	Euthanized	36	No Mets	No Mets	N/A	Unknown cause
28	5	Alive	979	No Mets	No Mets	N/A	N/A
29	5	Euthanized	224	76	0	148	Pulmonary Metastatic Disease
31	5	Euthanized	119	No Mets	No Chemo	N/A	Euthanized—unrelated to OS

## Data Availability

Data from this study will be available upon reasonable request to the corresponding author.

## Supplementary Materials

The following supporting information can be downloaded at: https://www.mdpi.com/article/10.3390/cancers18152511/s1, Supplemental Figure S1: Schematic of patient sample collection. Whole blood was collected from patients at 3 time points; enrollment, 1-,3-, or 5- days post histotripsy (DPH), and 2-weeks post limb amputation surgery, and peripheral blood mononuclear cells (PBMCs) were isolated via density gradient separation technique for further Analysis. Ablated (treated) and non-ablated (untreated) tumor regions were collected at the time of surgery for further analysis. Supplemental Figure S2: The differential gene expression changes observed in non-ablated and ablated tumor regions relative to treatment-naïve OS gene expression. Panel (A) 1 DPH group, Panel (B) 3 DPH Group, and panel (C) 5 DPH group. None of the observed changes were statistically significant, and further details can be found in Supplementary Table S2. Supplemental Figure S3: Characterization of circulating immune cell populations and ablation outcomes based on patient outcomes. Patients were categorized in “below MST”, “Expected MST” or ”Above MST” based on disease progression where expected MST represents the documented MST of 10-12 months for OS patients that receive definitive standard of care. (A) Circulating T-cells, (B) Circulating B-cells, (C) Circulating monocytes, (D) Inflammation score of treated tumor region, and (E) %viable cells in treated tumor region. Supplemental Table S1: Primer sequences used for peripheral monocyte genotyping. All primers were purchased from BioRad. Supplemental Table S2: Patient demographics. Supplemental Table S3: Gene tables with fold change values for tumor differential gene expression analysis with Nanostring Canine Immuno-Oncology panel ablated tumor region relative to paired non-ablated tumor region. Supplemental Table S4: Gene fold changes of non-ablated and ablated tumor regions relative to treatment naïve OS tumor samples for Nanostring Analysis.
