# Supplementary material for: Immunological and Clinical Outcomes Associated with Histotripsy Ablation in Spontaneously Occurring Canine Osteosarcoma; Veterinary Clinical Trial Results
Source: Cancers (Basel). 2026 Aug 5;18(15):2511. doi: 10.3390/cancers18152511 (PMC13465314; doi:10.3390/cancers18152511)
Supplement: Supplementary file 1 [file cancers-18-02511-s001.zip › cancers-4385196-supplementary.pdf]

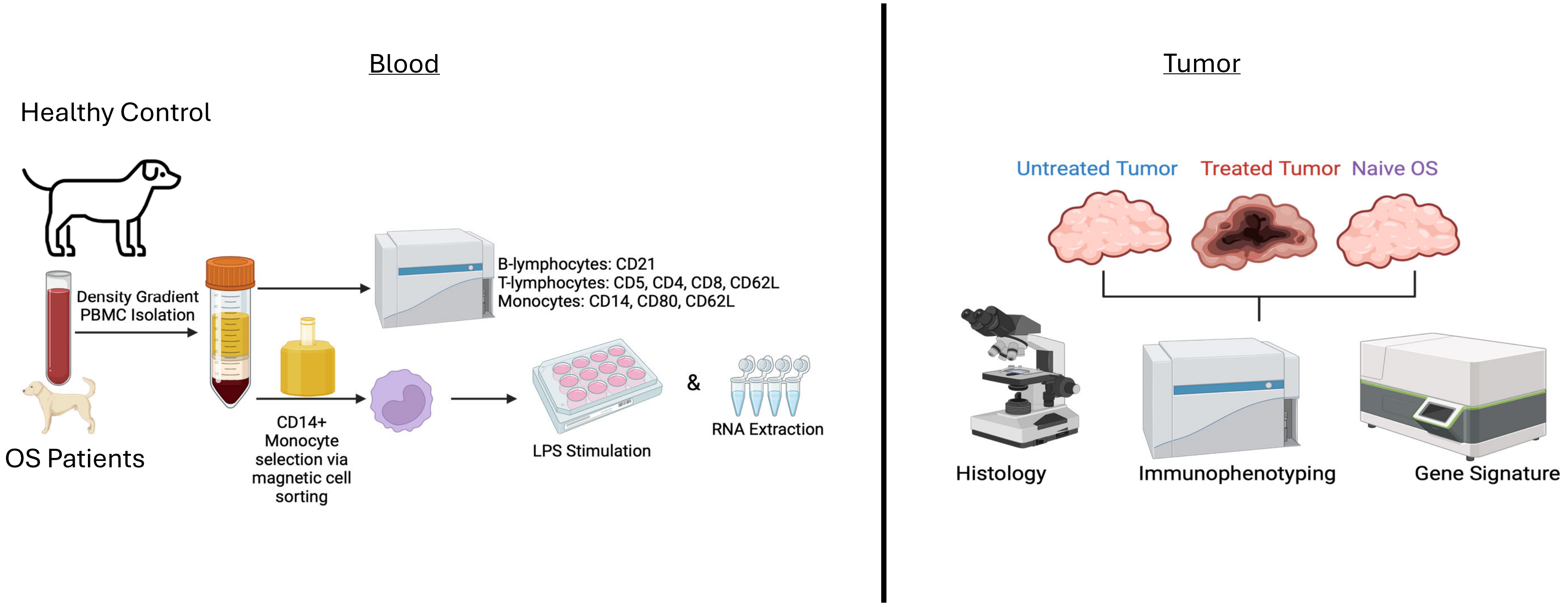

Supplemental Figure S1. Schematic of patient sample collection. Whole blood was collected from patients at 3 time points; enrollment, 1-,3-, or 5- days post histotripsy (DPH), and 2-weeks post limb amputation surgery, and peripheral blood mononuclear cells (PBMCs) were isolated via density gradient separation technique for further Analysis. Ablated (treated) and non-ablated (untreated) tumor regions were collected at the time of surgery for further analysis.

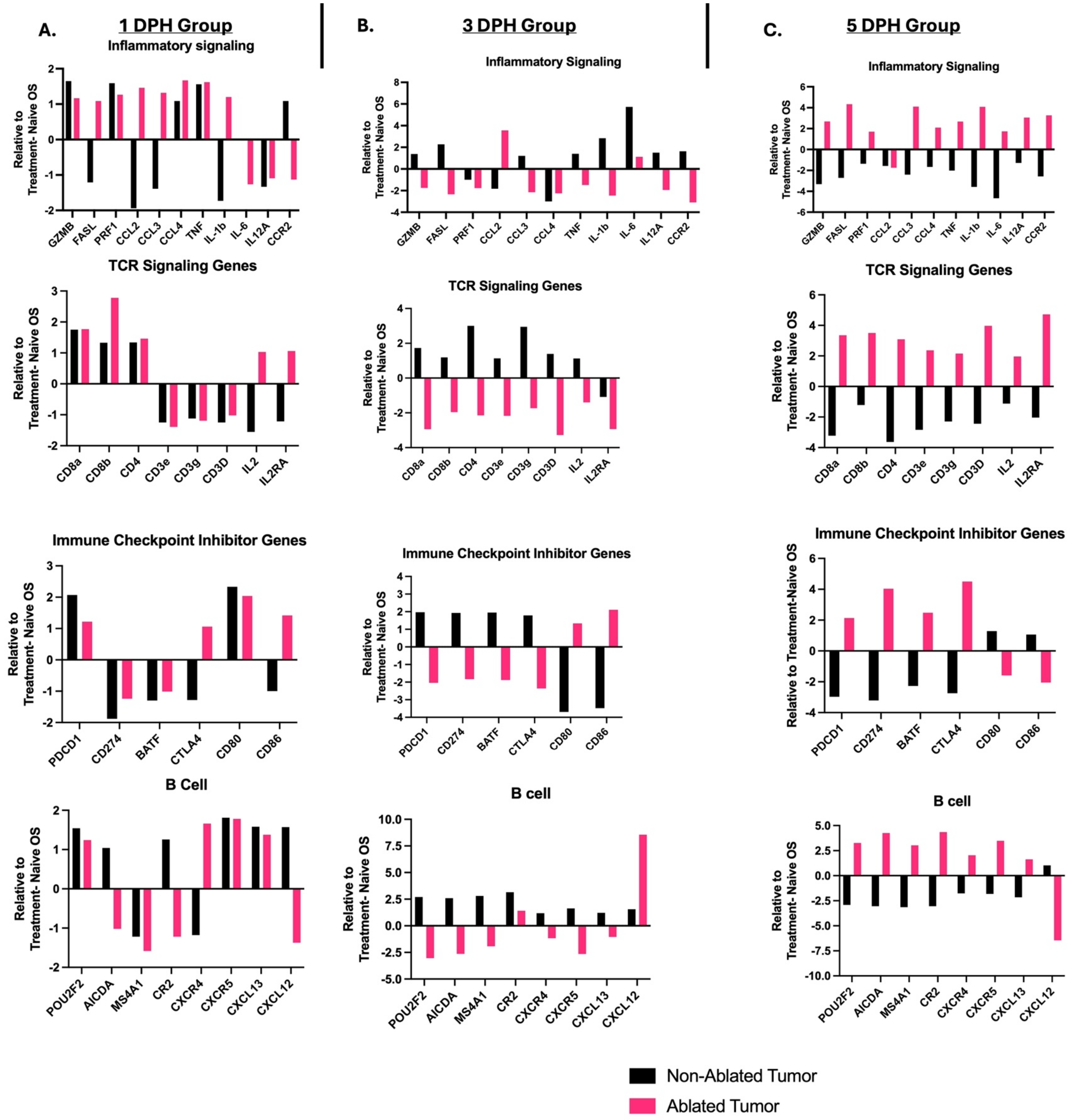

Supplemental Figure S2. The differential gene expression changes observed in non-ablated and ablated tumor regions relative to treatment-naïve OS gene expression. Panel (A) 1 DPH group, Panel (B) 3 DPH Group, and panel (C) 5 DPH group. None of the observed changes were statistically significant, and further details can be found in Supplementary Table S2.

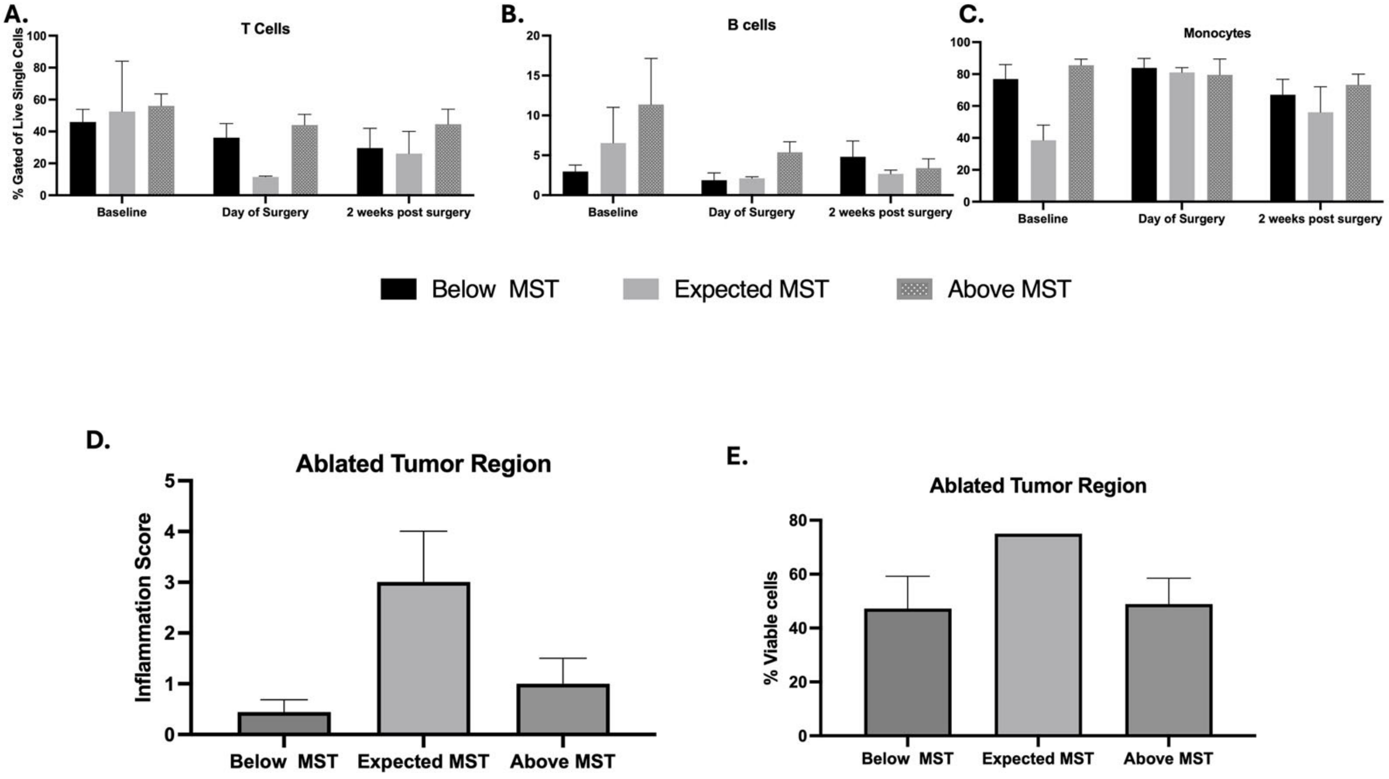

Supplemental Figure S3. Characterization of circulating immune cell populations and ablation outcomes based on patient outcomes. Patients were categorized in “below MST” , “Expected MST” or ”Above MST” based on disease progression where expected MST represents the documented MST of 10-12 months for OS patients that receive definitive standard of care. (A) Circulating T-cells, (B) Circulating B-cells, (C) Circulating monocytes, (D) Inflammation score of treated tumor region, and (E) %viable cells in treated tumor region.

Supplemental Table S1. Primer sequences used for peripheral monocyte genotyping. All primers were purchased from BioRad.

**Gene**	**Ensembl ID-Primer Sequence**
GAPDH	ENSCAFG00000007265
ACTB	ENSCAFG00000016020
IL10	ENSCAFG00000011443
IFNg	ENSCAFG00000000408
TNF	ENSCAFG00000000517

Supplemental Table S2. Patient demographics.

PATIENT	SURGERY GROUP*	BREED	AGE (YEARS)	GENDER	WEIGHT (KGS)	TUMOR PATHOLOGY
**1**	1	Malamute	6	MN	50	Osteosarcoma
**2**	1	Great Pyrenees	7.5	F	33	Osteosarcoma
**3**	1	Shepard Mix	2	MN	27	Osteosarcoma
**4**	1	Black Lab	10	FS	46	Osteosarcoma
**5**	1	Doberman	8	MN	45	Osteosarcoma
**6**	1	Rottweiler	7	MN	48	Osteosarcoma
**7**	1	Border Collie	9	FS	22	Osteosarcoma
**8**	1	Great Pyrenees	7	MN	57	Osteosarcoma
**9**	1	Mix	3	MN	17	Chondrosarcoma
**10**	1	Mix	8	MN	35	Osteosarcoma
**11**	3	Pit Bull	7	FS	22	Osteosarcoma
**12**	3	Shepard Mix	8	FS	38	Osteosarcoma
**13**	3	Bernese Mt. Dog	3	MN	40	Osteoma
**14**	3	Golden Retriever	6	MN	33	Osteosarcoma
**15**	3	St. Bernard	6	FS	77	Osteosarcoma
**16**	3	Husky	7	MN	32	Osteosarcoma
**17**	3	Greyhound	7	MN	37	Osteosarcoma
**18**	3	German Shepard	8	FS	37	Osteosarcoma
**19**	3	St. Bernard	3	MN	57	Osteosarcoma
**20**	3	Dachshund	13	MN	7	Hemangiosarcoma
**21**	3	Newfoundland	4	FS	42	Osteosarcoma
**22**	5	Pitbull	**11**	MN	28	Osteosarcoma
**23**	5	Mix	8	MN	50	Osteosarcoma
**24**	5	Boston Terrier	12	MN	15	Osteosarcoma
**25**	5	Great Pyrenees	8	FS	38	Chondrosarcoma
**26**	5	Husky Mix	9	FS	25	Osteosarcoma
**27**	5	Golden Retriever	11	MN	32	Osteosarcoma
**28**	5	Pointer	6	FS	32	Osteosarcoma
**29**	5	Poodle	8	MN	29	Osteosarcoma
**30**	5	Boxer	9	FS	26	Osteosarcoma
**31**	5	Cane Corso	9	FS	50	Osteosarcoma
**32**	5	Cane Corso	9	FS	50.5	Osteosarcoma
**NAÏVE PATIENT 1**	N/A	Leonberger	4	FS	60	Osteosarcoma
**NAÏVE PATIENT 2**	N/A	Labrador Retriever	9	M	37	Osteosarcoma
**NAÏVE PATIENT 3**	N/A	Mixed	8	FS	34	Osteosarcoma
**NAÏVE PATIENT 4**	N/A	Mixed	Unknown	FS	Unknown	Osteosarcoma
**NAÏVE PATIENT 5**	N/A	Pit Bill	10	FS	30	Osteosarcoma
**NAÏVE PATIENT 6**	N/A	Great Dane	9	MN	42.6	Osteosarcoma
**NAÏVE PATIENT 7**	N/A	Rottweiler	12	MN	32.8	Osteosarcoma
**NAÏVE PATIENT 8**	N/A	Mix Breed	15	FS	18	Osteosarcoma

* *Number of days post histotripsy* that limb amputation surgery occurred. MN= Male Neutered, FS= Female Spayed, F= Female. Naïve patients served as negative controls and did not receive tumor directed therapy prior to limb amputation surgery and tumor sample collection. *The histotripsy ablation outcomes and patient demographics for patients 1-10 were previously published (Ruger et al. 2023 [20]. Cancers). The histotripsy outcomes and demographics for patients 19, and 26-32 were previously published (Vickers et al. 2025 [25]. Ultrasound Medicine & Biology).*

Supplemental Table S3. Gene tables with fold change values for tumor differential gene expression analysis with Nanostring Canine Immuno-Oncology panel ablated tumor region relative to paired non-ablated tumor region.

A.	Ablated Tumor Relative to Non-ablated Tumor								
	1 DPH Group			3 DPH Group			5 DPH Group		
Gene Name	Fold Change	P-value	FDR	Fold Change	P-value	FDR	Fold Change	P-value	FDR
GZMB	-1.46	0.46	1	2.31	0.40	1	8.88	0.009	0.33
FASL	1.32	0.60	1	5.31	0.12	1	11.7	0.007	0.33
PRF1	-1.25	0.61	1	1.62	0.21	1	2.3	0.08	0.93
CCL2	2.83	0.09	1	-6.53	0.03	1	-1.04*	0.92	1
CCL3	1.84	0.25	1	2.59	0.35	1	9.89	0.01	0.34
CCL4	1.53	0.39	1	-1.43	0.7	1	3.47	0.04	0.54
TNF	1.03	0.93	1	2.07	0.17	1	5.4	0.005	0.33
IL-1β	2.07	0.27	1	6.97	0.1	1	14.6	0.009	0.33
IL-6	-1.01	0.99	1	5.11	0.12	1	8.09	0.01	0.34
IL12A	1.23	0.65	1	2.91	0.04	1	3.89	0.003	0.33
CCR2	-1.23	0.66	1	4.96	0.005	0.59	8.45	0.01	0.37

B.	Ablated Tumor Relative to Non-ablated Tumor								
	1 DPH Group			3 DPH Group			5 DPH Group		
Gene Name	Fold Change	P value	FDR	Fold Change	P-value	FDR	Fold Change	P-value	FDR
PDCD1	-1.7	0.32	1	4.02	0.01	0.89	6.35	0.04	0.6
CD274	1.51	0.48	1	3.53	0.24	1	12.96	0.003	0.33
BATF	1.23	0.42	1	3.66	0.08	1	5.62	0.06	0.71
CTLA4	1.25	0.56	1	2	0.21	1	12.4	0.003	0.33
CD80	-1.14	0.74	1	-4.93	0.01	0.92	-2.06	0.22	1
CD86	1.42	0.53	1	-7.43	0.08	1	-2.14	0.51	1

C.	Ablated Tumor Relative to Non-ablated Tumor								
	1 DPH Group			3 DPH Group			5 DPH Group		
Gene Name	Fold Change	P value	FDR	Fold Change	P-value	FDR	Fold Change	P-value	FDR
CD8a	1.02	0.97	1	5.1	0.053	1	10.8	0.01	0.37
CD8b	2.01	0.09	1	1.3	0.61	1	4.24	0.04	0.56
CD4	1.09	0.88	1	6.47	0.03	0.93	11.2	0.02	0.4
CD3e	-1.11	0.88	1	2.47	0.29	1	6.72	0.008	0.33
CD3g	-1.06	0.89	1	2.08	0.33	1	4.93	0.001	0.33
CD3D	1.23	0.76	1	4.58	0.07	1	8.69	0.002	0.3
IL2	1.24	0.3	1	1.19	0.1	1	2.26	0.04	0.57
IL2RA	1.29	0.65	1	2.27	0.18	1	9.64	0.006	0.33

D.	Ablated Tumor Relative to Non-ablated Tumor								
	1 DPH Group			3 DPH Group			5 DPH Group		
**Gene Name**	**Fold Change**	**P-value**	**FDR**	**Fold Change**	**P-value**	**FDR**	**Fold Change**	**P-value**	**FDR**
**POU2F2**	-1.25	0.7	1	8.29	0.002	0.35	9.56	0.009	0.34
**AICDA**	-1.06	0.9	1	6.93	0.008	0.77	12.96	0.005	0.33
**MS4A1**	-1.28	0.63	1	5.27	0.08	1	10.53	0.006	0.33
**CR2**	-1.53	0.5	1	7.3	0.09	1	13.14	0.004	0.33
**CXCR4**	1.96	0.02	1	1.38	0.56	1	3.62	0.0007	0.15
**CXCR5**	-1.02	0.95	1	4.3	0.05	1	6.35	0.005	0.33
**CXCL13**	-1.14	0.83	1	1.26	0.71	1	3.5	0.06	0.72
**CXCL12**	-2.16	0.41	1	-5.47	0.08	1	-6.64	0.11	1

FDR= False discovery ratio.

Supplemental Table S4. Gene fold changes of non-ablated and ablated tumor regions relative to treatment naïve OS tumor samples for Nanostring Analysis.

A. Inflammatory signaling	Ablated Relative to Treatment-Naive								
	1 DPH Group			3 DPH Group			5 DPH Group		
**Gene Name**	Fold Change	P value	FDR	Fold Change	P-value	FDR	Fold Change	P-value	FDR
**GZMB**	1.17	0.81	1	-1.74	0.54	1	2.68	0.24	1
**FASL**	1.09	0.92	1	-2.34	0.42	1	4.33	1.2	1
**PRF1**	1.27	0.60	1	-1.77	0.16	1	1.7	0.3	1
**CCL2**	1.46	0.5	1	3.56	0.02	1	-1.75	0.59	1
**CCL3**	1.32	0.72	1	-2.14	0.48	1	4.1	0.16	1
**CCL4**	1.67	0.35	1	-2.24	0.29	1	2.09	0.24	1
**TNF**	1.62	0.35	1	-1.48	0.56	1	2.67	0.19	1
**IL-1B**	1.2	0.84	1	-2.45	0.43	1	4.08	0.17	1
**IL-6**	-1.26	0.84	1	1.12	0.9	1	1.73	0.50	1
**IL12A**	-1.09	0.89	1	-1.94	0.3	1	3.05	0.10	1
**CCR2**	-1.13	0.85	1	-3.08	0.08	1	3.27	0.14	1

A. Inflammatory signaling	Non-ablated Relative to Treatment-Naive								
	1 DPH Group			3 DPH Group			5 DPH Group		
**Gene Name**	Fold Change	P value	FDR	Fold Change	P-value	FDR	Fold Change	P-value	FDR
**GZMB**	1.65	0.4	1	1.38	0.72	1	-3.32	0.09	1
**FASL**	-1.21	0.81	1	2.27	0.3	1	-2.72	0.25	1
**PRF1**	1.59	0.25	1	-1	0.5	1	-1.36	0.58	1
**CCL2**	-1.94	0.24	1	-1.83	0.4	1	-1.57	0.41	1
**CCL3**	-1.39	0.65	1	1.21	0.83	1	-2.41	0.32	1
**CCL4**	1.09	0.83	1	-3	0.19	1	-1.67	0.47	1
**TNF**	1.56	0.19	1	1.4	0.58	1	-2.02	0.31	1
**IL-1β**	-1.73	0.53	1	2.84	0.24	1	-3.58	0.17	1
**IL-6**	0	0	1	5.73	0.05	1	-4.67	0.09	1
**IL12A**	-1.33	0.65	1	1.5	0.48	1	-1.28	0.68	1
**CCR2**	1.09	0.88	1	1.63	0.4	1	-2.58	0.22	1

B. Immune Check Point Inhibitors	Ablated Relative to Treatment-Naïve								
	1 DPH Group			3 DPH Group			5 DPH Group		
**Gene Name**	Fold Change	P value	FDR	Fold Change	P-value	FDR	Fold Change	P-value	FDR
**PDCD1**	1.22	0.74	1	-2.04	0.26	1	2.14	0.37	1
**CD274**	-1.24	0.8	1	-1.83	0.56	1	4.04	0.12	1
**BATF**	-1.01	0.99	1	-1.88	0.41	1	2.48	0.31	1
**CTLA4**	1.06	0.95	1	-2.36	0.46	1	4.5	0.13	1
**CD80**	2.04	0.10	1	1.34	0.62	1	-1.59	0.48	1
**CD86**	1.42	0.37	1	2.11	0.42	1	-2.05	0.51	1

**B. Immune Check Point Inhibitors**	**Non-Ablated Relative to Treatment-Naïve**								
	1 DPH Group			3 DPH Group			5 DPH Group		
**Gene Name**	Fold Change	P value	FDR	Fold Change	P-value	FDR	Fold Change	P-value	FDR
**PDCD1**	2.07	0.22	1	1.97	0.28	1	-2.97	0.13	1
**CD274**	-1.88	0.45	1	1.93	0.42	1	-3.21	0.18	1
**BATF**	-1.3	0.57	1	1.95	0.27	1	-2.27	0.25	1
**CTLA4**	-1.28	0.76	1	1.79	0.53	1	-2.75	0.29	1
**CD80**	2.33	0.03	1	-3.69	0.03	1	1.29	0.69	1
**CD86**	-1	0.99	1	-3.48	0.03	1	1.06	0.93	1

**C. TCR Signaling**	**Ablated Relative to Treatment- Naïve**								
	1 DPH Group			3 DPH Group			5 DPH Group		
Gene Name	Fold Change	P value	FDR	Fold Change	P-value	FDR	Fold Change	P-value	FDR
**CD8a**	1.77	0.48	1	-2.94	0.21	1	3.35	0.21	1
**CD8b**	2.78	0.19	1	-1.96	0.45	1	3.5	0.19	1
**CD4**	1.46	0.65	1						
**CD3e**	-1.39	0.72	1	-2.17	0.32	1	2.37	0.25	1
**CD3g**	-1.19	0.78	1	-1.73	0.82	1	2.15	0.17	1
**CD3D**	-1.02	0.98	1	-3.28	0.09	1	3.97	0.06	1
**IL2**	1.03	0.96	1	-1.4	0.39	1	1.96	0.14	1
**IL2RA**	1.06	0.95	1	-2.93	0.23	1	4.72	0.1	1
**C. TCR Signaling**	**Non-Ablated Relative to Treatment-Naïve**								
	1 DPH Group			3 DPH Group			5 DPH Group		
**Gene Name**	Fold Change	P value	FDR	Fold Change	P-value	FDR	Fold Change	P-value	FDR
**CD8a**	1.75	0.45	1	1.73	0.54	1	-3.22	0.19	1
**CD8b**	1.33	0.67	1	1.19	0.84	1	-1.21	0.81	1
**CD4**	1.34	0.7	1						
**CD3e**	-1.25	0.77	1	1.14	0.88	1	-2.84	0.19	1
**CD3g**	-1.12	0.69	1	2.95	0.15	1	-2.29	0.13	1
**CD3D**	-1.25	0.74	1	1.39	0.66	1	-2.44	0.19	1
**IL2**	-1.55	0.38	1	1.13	0.74	1	-1.12	0.71	1
**IL2RA**	-1.21	0.85	1	-1.08	0.93	1	-2.04	0.43	1

**D. B cell**	**Ablated Tumor Relative to Naïve Tumor**								
	**1 DPH Group**			**3 DPH Group**			**5 DPH Group**		
**Gene Name**	**Fold Change**	**P-value**	**FDR**	**Fold Change**	**P-value**	**FDR**	**Fold Change**	**P-value**	**FDR**
**POU2F2**	1.24	0.76	1	-3.13	0.16	1	3.27	0.21	1
**AICDA**	-1.02	0.98	1	-2.63	0.22	1	4.26	0.11	1
**MS4A1**	-1.58	0.57	1	-1.92	0.5	1	3.03	0.19	1
**CR2**	-1.22	0.83	1	1.42	0.3	1	4.35	0.14	1

**CXCR4**	1.66	0.15	1	-1.17	0.75	1	2.04	0.05	1
**CXCR5**	1.78	0.27	1	-2.64	0.19	1	3.48	0.1	1
**CXCL13**	1.38	0.62	1	-1.04	0.96	1	1.63	0.56	1
**CXCL12**	-1.37	0.78	1	8.56	0.09	1	-6.46	0.15	1
**D. B-cell**	**Non-ablated Tumor Relative to Naïve Tumor**								
	**1 DPH Group**			**3 DPH Group**			**5 DPH Group**		
**Gene Name**	**Fold Change**	**P-value**	**FDR**	**Fold Change**	**P-value**	**FDR**	**Fold Change**	**P-value**	**FDR**
**POU2F2**	1.54	0.53	1	2.7	0.3	1	-2.92	0.22	1
**AICDA**	1.04	0.96	1	2.6	0.19	1	-3.04	0.17	1
**MS4A1**	-1.22	0.79	1	2.8	0.17	1	-3.14	0.11	1
**CR2**	1.26	0.79	1	3.15	0.21	1	-3.04	0.25	1
**CXCR4**	-1.18	0.60	1	1.18	0.74	1	-1.77	0.09	1
**CXCR5**	1.81	0.24	1	1.63	0.4	1	-1.82	0.38	1
**CXCL13**	1.58	0.54	1	1.22	0.9	1	-2.15	0.31	1
**CXCL12**	1.57	0.62	1	1.56	0.61	1	1.03	0.97	1
